# Single-gate electro-optic beam switching metasurfaces

**DOI:** 10.1038/s41377-025-01967-y

**Published:** 2025-08-27

**Authors:** Sangjun Han, Jinseok Kong, Junho Choi, Won Chegal, Min Seok Jang

**Affiliations:** 1 School of Electrical Engineering, Korea Advanced Institute of Science and Technology, Daejeon, 34141 Republic of Korea; 2 Department of Mechanical Engineering, University of California, Berkeley, Berkeley, CA 94720 USA; 3 Strategic Technology Research Institute, Korea Research Institute of Standards and Science, Daejeon, 34113 Republic of Korea; 4 Department of Physics, Kyung Hee University, Seoul, 02447 Republic of Korea

**Keywords:** Metamaterials, Optical properties and devices, Mid-infrared photonics

## Abstract

Electro-optic active metasurfaces have attracted attention due to their ability to electronically control optical wavefronts with unprecedented spatiotemporal resolutions. In most studies, such devices require gate arrays composed of a large number of independently-controllable local gate electrodes that address the local scattering response of individual metaatoms. Although this approach in principle enables arbitrary wavefront control, the complicated driving mechanism and low optical efficiency have been hindering its practical applications. In this work, we demonstrate an active beam switching device that provides highly directional beam profiles and significant and uniform optical efficiencies across diffraction orders separated by a large deflection angle. The device operates with only a single-gate bias applied to monolayer graphene, modulating its optical conductivity to control the optical efficiency of the device. The key performance metrics, the absolute and the relative efficiency, which are defined as the scattered power toward a certain angle *θ* normalized by the incident power and the net scattered power from the metasurface, respectively, are maximized by a genetic algorithm. Experimentally, the metasurface achieves 57° of active beam switching from the 0th to the −1st order diffraction, with absolute efficiencies of 0.084 and 0.078 and relative efficiencies of 0.765 and 0.836, respectively. Furthermore, an analytical framework using nonlocal quasinormal mode expansion provides deeper insight into the operating mechanism of active beam switching. Finally, we discuss the performance limitations of this design platform and provide insights into potential improvements.

## Abstract (web summary)

We experimentally demonstrated and analyzed a single-gate electro-optic metasurface achieving efficient, wide-angle optical beam switching.

## Graphical abstract

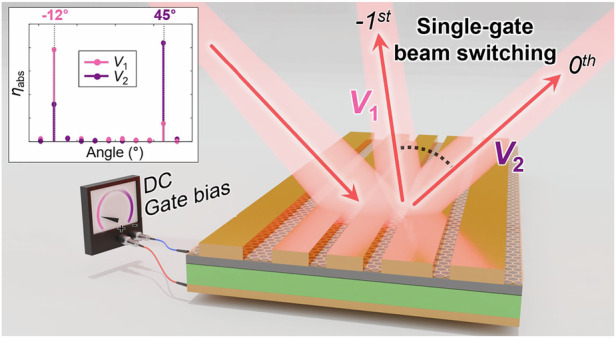

## Introduction

Dynamic control of optical beam direction is an emerging technology in a wide range of applications, including light detection and ranging (LiDAR), freespace optical communication, laser display, and laser machining. Conventional beam control methods that rely on mechanically moving parts^1^ have often suffered from their bulky size and significant power consumption. MEMS-based beam control devices combining an actuator and a microscanner^2^ have been adopted to solve this problem. However, these approaches still suffer from low durability and operation speed.

Active metasurfaces provide a promising route to overcome these challenges as they enable a precise control of the wavefront of light with unprecedented spatiotemporal resolutions^3–8^. Active beam switching metasurfaces have been implemented utilizing various active tuning mechanisms, including mechanical^9–14^, thermal^15–19^, and electric^20,21^ methods. Among these tuning mechanisms, electrically tunable beam switching method based on electro-optic materials such as indium tin oxide^7,22,23^, metallic polymer^24^ or transition metal dichalcogenides^25^ is attracting attention as it offers a small device footprint, reduced power consumption, minimal heat generation, and improved operation speed over other tuning mechanisms^1,26,27^. Injecting carriers into the electro-optic materials alters their refractive indices, modulating the optical response of the metasurface. To overcome small electro-optic index change and maximize the optical modulation of the device, most electro-optic metasurfaces leverage optical resonances to enhance interaction between light and matter^28–30^. In recent years, electro-optic metasurfaces have been demonstrated to have active full-2*π* phase modulation with a nearly constant amplitude by employing two or more tuning parameters per metaatom^6,22^ or avoided crossing of two resonances^31^ to break the strong correlation between the phase and amplitude response of a resonance. To implement a desired spatial wavefront, individual metaatoms need to be independently gated based on their location in a metasurface with a large number of local gate electrodes^7,22^.

Although these approaches in principle enable arbitrary wavefront manipulation, the devices based on local resonance control of metaatoms require complex circuit drivers to individually address the scattering response of each metaatom. This complex driving mechanism can cause potential malfunctions and hinder miniaturization. Furthermore, due to their large losses caused by strong light-matter interaction, these approaches tend to exhibit reduced optical efficiencies^22,24,25^. Moreover, large-angle beam deflection, which requires a steep spatial phase ramp, is particularly challenging for the devices using unit-cell design approaches due to the crosstalk between the neighboring metaatoms^32^. Consequently, these devices typically exhibit uneven efficiencies across multiple diffraction orders. To make beam switching technology more practical, the challenge remains to simplify the driving mechanism while improving the performance. However, solving the aforementioned problems with a single tuning parameter is a formidable design challenge for the electro-optic metasurfaces, even two-level electro-optic beam switching metasurfaces driven by a single tuning parameter have not yet been experimentally demonstrated.

In this study, we design and demonstrate an efficient electro-optic beam switching device that operates with a single global gate instead of an array of numerous local gate electrodes. This metasurface simultaneously exhibits high relative efficiency, uniform and significant absolute efficiency comparable to the performance of state-of-the-art conventional electro-optic beam steering metasurfaces^7,22–25^, and a high deflection angle while operating with only a single-gate bias in the mid-infrared region. The key performance metrics, the absolute and the relative efficiency, are defined as the scattered power toward a certain angle *θ* normalized by the incident power, and the net scattered power from the metasurface, respectively (see Supplementary Note 1). To tackle the structural design challenge to get a high performance even with a single tuning parameter, we apply an inverse design using the genetic algorithm rather than adopting a unit-cell design approach based on a locally periodic approximation (LPA). Quasinormal mode (QNM) analysis reveals that the device operation is based on the interference between a gate-tunable resonant mode and a non-resonant background response. Finally, we show that the same design principle can be applied to achieve high-efficiency multi-level beam switching theoretically. Our work constitutes a stepping stone towards reliable dynamic optical beam control.

## Results

### Device geometry and fabrication

**Fig. 1:**
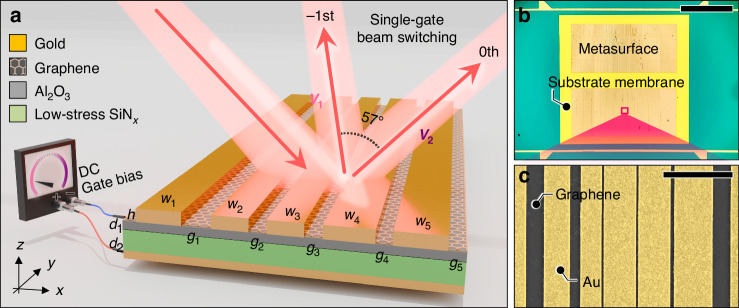
Single-gate electro-optic beam switching metasurfaces. **a** Schematic of an active beam switching graphene metasurface with gold strip width (*w*_1_, *w*_2_, *w*_3_, *w*_4_, *w*_5_) = (1135, 914, 1403, 1387, 1671) nm, gap (*g*_1_, *g*_2_, *g*_3_, *g*_4_, *g*_5_) = (433, 71, 71, 142, 733) nm, gold strip height *h* = 64 nm, Ti adhesion layer thickness 6 nm, Al_2_O_3_ thickness *d*_1_ = 30 nm, and SiN_*x*_ thickness *d*_2_ = 200 nm. The DC gate bias is applied between the gold back reflector and monolayer graphene. The operation frequency is *f*_0_ = 41.17 THz. **b** Optical microscope top-view image of the fabricated device. Two rectangular regions with deposited gold gratings are located on a substrate membrane, which serves as a dielectric layer. To apply a single-gate bias to the graphene layer, two electrode lines are positioned above and below the substrate membrane. The scale bar is 400 μm. **c** Scanning electron microscope (SEM) top-view image of the grating for one period (false colored). The yellow area indicates gold strips, and the black area indicates gaps where graphene is exposed. The scale bar is 3 μm

The active beam switching graphene metasurface is designed and fabricated through several intricate steps to achieve the desired optical performance in the mid-infrared regime. A schematic of the proposed device is illustrated in Fig. 1a. TM-polarized light incident at *θ*_inc_ = 45° is reflected by the metasurface with grating period *P* and diffracted to either the 0th or −1st order channel, depending on the global gate bias applied to the monolayer graphene. We set *P* = 7.960 μm to enable only 0th and −1st order diffraction channels at the operation frequency *f*_0_ = 41.17 THz (*λ*_0_ = 7.281 μm) while suppressing all the higher order diffractions. The −1st order diffraction angle *θ*_−1_ = −11.98° is determined by sin *θ*_−1_ = sin *θ*_inc_−*λ*_0_/*P*. The topmost layer of the metasurface consists of a periodic gold grating, which scatters incident light into 0th or −1st order diffraction channels. A single grating period is composed of five gold strips, each of which has a width (*w*_*i*_) and gap (*g*_*i*_) between the neighboring elements. In the gaps between gold strips, monolayer graphenes cover the exposed substrate as shown in Fig. 1a. The substrate is a 200 nm low-stress silicon nitride membrane with a 30 nm thin film of aluminum oxide deposited on top. This thin alumina layer plays a crucial role in increasing the stability of the device by suppressing gate leakage current^33,34^ (see Supplementary Note 2). On the backside of the device, a 70 nm gold layer with a 3 nm Ti adhesion layer serves as a global back gate electrode and also as a back reflector to block transmission channels^28–30^.

The key principle of beam switching relies on Fermi level modulation of graphene via the global back gate bias *V*_G_ applied between the back reflector and the graphene, which alters the absolute efficiency of each order^35^. As the Fermi level of graphene switches between the charge neutrality point (CNP) and 0.42 eV, the surface conductivity of the graphene is modified correspondingly^36^. These two Fermi levels are selected to maximize beam switching performance within the stable operation range bounded by the dielectric strength of the gate dielectrics.

Figure 1b shows an overall view of the fabricated device taken under an optical microscope. The substrate membrane is supported by a surrounding thick silicon frame to facilitate handling of the entire chip. Two straight electrode lines are patterned on the silicon frame, positioned on either side of the membrane. The graphene layer extends over both the membrane and the silicon frame, forming electrical contacts with these electrode lines. This configuration enables the application of a gate bias and the measurement of source-drain current through the graphene channel using the patterned electrodes. The leakage current and electrical stability of the gating scheme are further discussed in Supplementary Fig. S1b. The results demonstrate marginal hysteresis and robust endurance under cyclic gate voltage sweeps between −80 and +70 V.

Our metasurface is fabricated through the following steps. With the silicon nitride membrane prepared, a gold back reflector is deposited on the backside using a thermal evaporator. Then, a thin aluminum oxide layer is deposited on the front side of the membrane via atomic layer deposition (ALD). Once the dielectric layers are set up, monolayer graphene is transferred onto the aluminum oxide layer using a wet-transfer technique. Electron beam lithography is performed on the graphene layer to specify the location where gold is deposited. To ensure good adhesion of the deposited gold layer, the exposed part of the graphene is etched with an oxygen plasma asher. The device is completed by forming gold gratings through a lift-off process after depositing gold with a thermal evaporator. Detailed fabrication steps are provided in the “Methods” section. Figure 1c provides a top-view SEM image of the gold grating for one period, confirming that the gold grating and graphene ribbons are clearly defined.

### Single-gate electro-optic beam switching in the mid-infrared regime

**Fig. 2:**
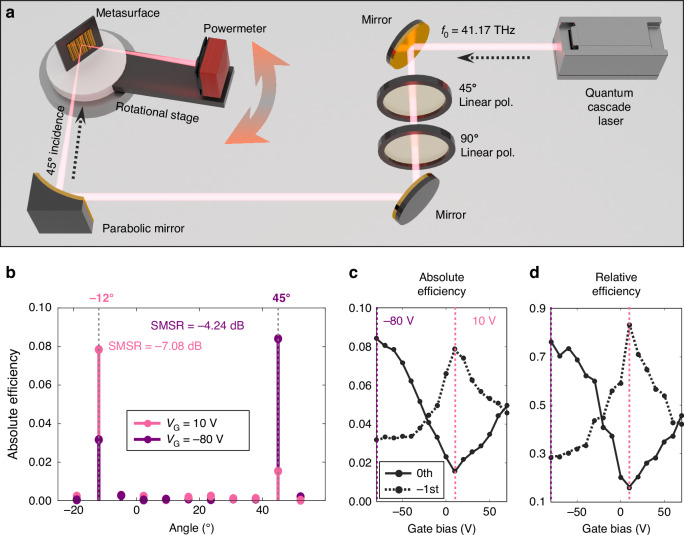
Optical setup and measured results. **a** Schematic of the optical setup. **b** Angle-resolved far-field pattern measured at the gate bias *V*_G_ = 10 V and *V*_G_ = −80 V at the operation frequency *f*_0_ = 41.17 THz. At *V*_G_ = 10 V (pink), the highest absolute efficiency is measured at −12°, which corresponds to the −1st order diffraction angle, and at *V*_G_ = −80 V (purple), the highest absolute efficiency is measured at 45°, which corresponds to the 0th order diffraction angle. **c** Experimentally measured absolute efficiencies and **d** relative efficiencies for the 0th and the −1st order diffractions when the gate bias is swept from *V*_G_ = −80 V to *V*_G_ = 70 V

The performance of the single-gate active beam switching is evaluated using an optical setup depicted in Fig. 2a. The experimental setup is built to precisely measure the absolute efficiency of the reflected light from the metasurface over a wide angular range. Initially, light from the quantum cascade laser is polarized into the TM mode by passing through the 45° and 90° polarizers sequentially, and then is focused onto the metasurface using a parabolic mirror. The absolute efficiency of the reflected light is measured with a power meter mounted on the rotational stage, allowing the observation of diffracted light at various angles. Detailed information of the optical measurement is described in the “Methods” section.

The angle-resolved far-field pattern is measured at each gate bias *V*_G_ at the operation frequency of *f*_0_ = 41.17 THz by rotating the stage where the power meter is mounted, to characterize the quality of the diffracted beam as shown in Fig. 2b. The side mode suppression ratio is also assessed, yielding values of −7.08 dB for the −1st order diffraction at *V*_G_ = 10 V and −4.24 dB for the 0th order diffraction at *V*_G_ = −80 V, meaning that the light scattered by the metasurface is highly directed into the two diffraction channels with marginal side lobes. The angular interval between measurement points is determined by the angular field of view of the power meter.

The beam switching capability of the fabricated device is further characterized by measuring the optical efficiencies as a function of the gate bias at the two angular peak positions where the 0th and −1st order diffractions mainly occur. The gate bias is swept from −80 to 70 V. At *V*_G_ = 10 V, which corresponds to the CNP, the metasurface exhibits the highest absolute efficiency of 0.078 at −12° (Fig. 2c). This major angle is consistent with the theoretical −1st order diffraction angle, calculated based on the period of the metasurface measured by Atomic Force Microscope. In contrast, at *V*_G_ = −80 V, the major angle shifts to 45°, which is the 0th order diffraction (specular reflection) angle, with the absolute efficiency of 0.084. The relative efficiencies of each diffraction order are calculated by taking the ratio of the absolute efficiency at each diffraction angle to the total reflected power over the entire measurable angular space. Figure 2d shows the active tuning of the relative efficiency for each diffraction order as a function of the gate bias. At *V*_G_ = 10 V, the relative efficiency of the −1st order diffraction is 0.836, and the relative efficiency of the 0th order diffraction at *V*_G_ = −80 V is 0.765. Noteworthily, as the gate bias changes from −80 to 10 V, it can be seen that the relative efficiencies of the 0th and −1st order diffractions are gradually crossing, which is the characteristic that could potentially be used as a tunable freespace beam splitter.

### Device design and analysis based on electromagnetic simulation

The structure parameters and the operation frequency of our metasurface are optimized using a genetic algorithm. A genetic algorithm is an optimization method that mimics the process of natural selection in biological evolution, and it is a classic but well-known for its powerful performance^37,38^. Since it is not gradient-based, it can optimize even discontinuous and complicated figures of merit and has been widely utilized to optimize the structure of metasurfaces^39–42^. To design a metasurface with high beam switching performance, we set the figure of merit to simultaneously maximize the absolute and the relative efficiency in the 0th order diffraction at one target Fermi level, and in the −1st order diffraction at the other target Fermi level. Each gene in the gene pool is characterized by the gold strip widths *w*_*i*_, gap sizes *g*_*i*_, the metasurface height *h*, and the target operation frequency *f*_0_. Here, *i* represents the index of the gold strip (1 ≤ *i* ≤ 5). The optimized structure and the operation frequency are obtained by iterating the selection, cross-over, and mutation processes within the gene pool until the figure of merit is saturated at a certain extremum point^37^. Detailed figure of merit, design parameters, and an optimization flow chart are described in the Supplementary Note 3.

The actual fabricated device exhibits structure parameters that deviate slightly from the optimal values due to minor fabrication errors. However, our design platform has a high tolerance for these errors, so the figure of merit is not significantly degraded (see Supplementary Note 4). The operation frequency for our experiment is chosen as a slightly shifted value from the design operation frequency based on the highest calculated figure of merit spectrum. This frequency provides an optimal balance between the absolute and the relative efficiency, making it ideal to demonstrate the beam switching capability of the metasurface.

**Fig. 3:**
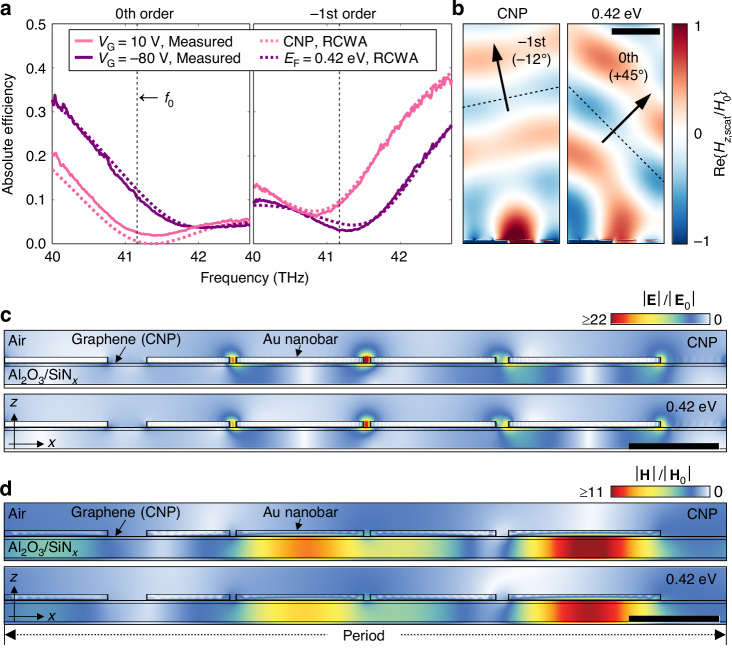
Electromagnetic simulation of the single-gate electro-optic beam switching metasurfaces. **a** Experimentally measured (solid lines) and electromagnetically simulated (dashed lines) absolute efficiency spectra for the 0th and the −1st order diffractions. **b** Simulated scattered magnetic field profile, **c** electric field intensity profile, and **d** magnetic field intensity profile of the charge neutrality point (CNP) and *E*_F_ = 0.42 eV at the operation frequency *f*_0_ = 41.17 THz. The scale bars are 4 μm in (**b**) and 1 μm in (**c**, **d**)

To understand the mechanism underlying the active beam switching of the optimized metasurface structure and to validate the experimental results, a detailed numerical analysis is performed using the rigorous coupled wave analysis (RCWA)^43–45^. As shown in Fig. 3a, the absolute efficiency spectra for both the 0th and −1st order diffractions under gate biases *V*_G_ of 10 V and −80 V are closely consistent with the experimental data, confirming the reliability of the simulation framework. As the gate bias approaches −80 V from the CNP of *V*_G_ = 10 V, the graphene becomes more conducting (i.e., the real part of graphene permittivity becomes more negative), resulting in a blueshift of diffraction spectra as predicted by the first-order perturbation theory^31,46,47^. Based on a simple parallel plate capacitor model and the measured CNP from the electrical transport measurement, the gate bias swing from 10 to −80 V is converted to the graphene Fermi level *E*_F_ swing from CNP (0 eV) to 0.42 eV^35^, and these values are used in the simulation. A graphene carrier mobility is assumed to be 200 cm^2^/V⋅s, which shows the best agreement with the experimental results and is consistent with previously observed values at mid-infrared frequencies^30^. This low graphene carrier mobility can be attributed to the impurities induced during the wet-transfer process and O_2_ plasma ashing used in fabricating graphene ribbons^48^, but it is not a major problem as the device is designed to be robust under carrier mobility degradation (see Supplementary Note 4). At the CNP, the 0th order diffraction spectrum is calculated to have a near-zero minimum, whereas the measured spectrum has a nonzero finite minimum point. This discrepancy can be attributed to factors such as spatial inhomogeneity of the structure over the illuminated area and the presence of residual charges even in the CNP^49^ (see Supplementary Notes 1 and 5).

Figure 3b presents the simulated scattered magnetic field profile at the operation frequency for two Fermi levels (CNP and 0.42 eV). At these Fermi levels, the metasurface exhibits a deflection angle of 57°, shifting from the −1st order diffraction (−12°) to the 0th order diffraction (45°). Both switching states exhibit clear wavefronts with uniform absolute efficiency and high relative efficiency toward the target diffraction order. At the CNP, the wavefront is primarily directed toward the −1st order diffraction with the absolute efficiency of 0.097 and the relative efficiency of 0.949. At *E*_F_ = 0.42 eV, the wavefront switches toward the 0th order diffraction with the absolute efficiency of 0.122 and the relative efficiency of 0.723, showing high-performance active beam switching in mid-infrared.

The calculated electromagnetic field intensity profiles around the metasurface for these two switching states are presented in Fig. 3c, d. In both switching states, the electric field is concentrated in the narrower gaps between the gold strips since gold has a high electrical conductivity at mid-infrared frequencies, and thus the electric potential drop mostly occurs in the gap region where the graphene is exposed. The magnetic field is mostly confined within the dielectric layer between the back reflector and the graphene, spreading over a period of the metasurface. Interestingly, despite the significant change in the far-field wavefront, the intensity distribution of both electric and magnetic fields in the device remains similar regardless of the switching states. From the electromagnetic field distribution that is not tightly bound to the graphene or strongly dependent on *E*_F_, it is evident that the operation of our device is not relying upon graphene plasmon resonances, which have been utilized in many previous works to modulate mid-infrared light^6,28–30^.

It is important to note that the LPA fails to explain the operation of our device, which implies that interactions between gold strips play a pivotal role in device operation. We investigate the phase and amplitude responses of subunits (gold strips and gaps) composing the metasurface with LPA as detailed in Supplementary Note 6. Ideally, a metasurface designed with LPA should exhibit spatially uniform scattering amplitudes and spatially increasing scattering phases^50^. Additionally, in the active metasurface, the spatial gradient of the scattering phase should be tunable with control parameters. However, we find that the reflective phase gradient does not exhibit the mentioned behavior, indicating that our device should be understood beyond LPA.

### Quasinormal mode analysis

**Fig. 4:**
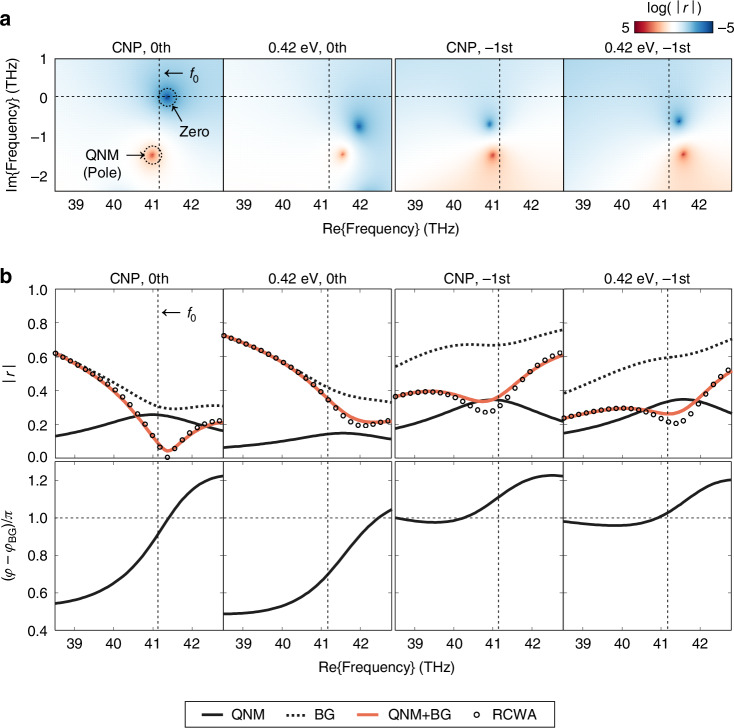
Quasinormal mode (QNM) analysis. **a** Spectra extended to the complex frequency of the log(|*r*|) for the 0th and the −1st order diffractions at the charge neutrality point (CNP) and *E*_F_ = 0.42 eV. Positively diverging points are the “poles” of the system, and negatively diverging points are the “zeros” of the system. **b** Complex diffraction coefficient |*r*|exp(i*φ*) spectra for the 0th and the −1st order diffractions at the CNP and *E*_F_ = 0.42 eV. In the upper panel, Reflection amplitude |*r*| spectra are decomposed with resonant QNM and non-resonant background response. Reconstructed reflection amplitude spectra (red solid lines) show good agreement with the electromagnetically simulated spectra (black circles). The lower panel shows the reflection phase difference with the background response *φ-φ*_BG_ spectra

To gain deeper insights into the operation mechanism of the presented device, we perform QNM analysis. A QNM represents the resonant state in the open and non-Hermitian system, which decays over time with a complex eigenfrequency^51^. We can understand the overall optical response of the metasurface as an interference between resonant QNMs and non-resonant background response^52–55^. Figure 4a shows the reflection amplitude in the complex frequency plane at the two representative Fermi levels. Around the operation frequency *f*_0_, we identify one positively diverging “pole,” which is the eigenfrequency of the QNM of the system. As the Fermi level increases, the eigenfrequency of the QNM blueshifts with decreasing graphene permittivity^31,46,47^ (see Supplementary Note 7). This tendency is also consistent with the blueshift of the entire spectrum in Fig. 3a, which is the trajectory line along the real frequency axis. At the same graphene Fermi level, the eigenfrequency of the QNM is the same regardless of the diffraction order. However, it contributes differently to the 0th and the −1st order diffraction spectrum along the real frequency axis. The QNM interferes with the non-resonant background response to produce “zeros” in Fig. 4a. Unlike the eigenfrequency or poles, these zeros are located differently across diffraction orders, even at the same Fermi level. In particular, for the 0th order diffraction at the CNP, the zero lies on the real frequency axis, which means nearly vanishing specular reflection. On the other hand, for the −1st order diffraction at the same Fermi level, the zero is far from the real frequency axis, resulting in the baseline of the reflection spectrum larger than 0. This order-specific difference accounts for the high relative efficiency of the wavefront toward the −1st order diffraction at the CNP.

We then decompose the complex diffraction coefficients into the individual contributions of the resonant QNM and non-resonant background response by employing the Reisz projection method^55^ as shown in Fig. 4b. As expected, the optical response by the QNM (black solid lines) clearly blueshifts with increasing |*E*_F_| (i.e., increasing carrier concentration). The background response (black dotted lines) presents non-resonant spectral behavior that marginally depends on *E*_F_. The small dependence of the background response on *E*_F_ is attributed to the additional QNMs located outside of the Reisz projection contour, far from the real operation frequency (see Supplementary Note 7). The complex sum of the QNM and the background response (red solid lines) agrees well with the RCWA-calculated spectrum (black circles), confirming the reliability of the decomposition. The decomposed electromagnetic field profiles of the QNM and the non-resonant background response are plotted in the Supplementary Figs. S13 and S14. The total electromagnetic field can be reconstructed by algebraically summing the decomposed field profiles. The reconstructed total field shows good agreement with the results in Fig. 3c, d, which are directly calculated utilizing RCWA under oblique incident plane waves.

The active beam switching behavior of the metasurface can be explained as an interference between the QNM and the background responses. At the CNP, for the 0th order diffraction, they have similar amplitudes and are out of phase, resulting in a nearly perfect destructive interference with a vanishing specular reflection at the operation frequency. In contrast, for the −1st order diffraction, the QNM shows much smaller amplitude compared to the background response. This mismatch in amplitude causes an incomplete cancellation, leading to a significant diffraction amplitude along the −1st order channel. Consequently, the wavefront at the CNP is predominantly directed toward the −1st order diffraction. Conversely, at *E*_F_ = 0.42 eV, the diffraction amplitude difference between the QNM and the background modes remains similar across diffraction orders. However, the phase difference for the −1st order diffraction is much closer to *π* than that of the 0th order diffraction, leading to wavefronts predominantly directed toward the 0th order diffraction.

## Discussion

**Fig. 5:**
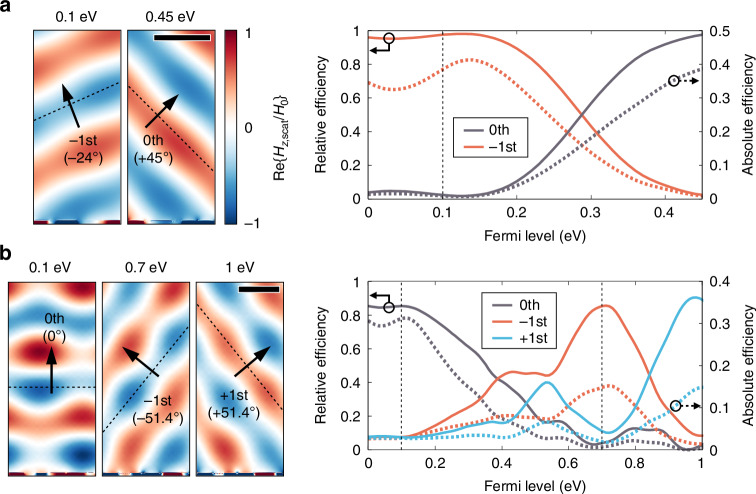
Optimization result of the active beam switching metasurfaces under relaxed constraints. Simulated scattered magnetic field profiles (left panel) and beam switching performance as a function of Fermi level (right panel) **a** for 2-level switching and **b** for 3-level switching. In the right panel, solid lines represent the relative efficiency (left axis) and dotted lines represent the absolute efficiency (right axis). The scale bars are 4 μm

To further explore the performance potential of the proposed platform, we optimize the structure with more relaxed fabrication and material quality constraints (see Supplementary Note 8). Here, the minimum feature size of the device structure is set to 20 nm, which can be achieved with advanced electron beam lithography techniques^56,57^. For this optimization, we assumed a graphene carrier mobility of 1000 cm^2^/V⋅s, which is readily achievable with CVD-grown graphene in practical applications^58^. Compared to the previous structure designed with tighter fabrication constraints, the newly optimized metasurface with the grating period of 6.097 μm, exhibits a significantly higher theoretical performance at the operation frequency of 44.16 THz as shown in Fig. 5a. For both the 0th and −1st order diffractions, a high absolute efficiency of 0.385 with near-unity relative efficiency of 0.976 is obtained, showing a clear wavefront in Fig. 5a.

Under these relaxed structural constraints, three-level active beam switching, including an additional channel for the 1st order diffraction, is also feasible. To accommodate three diffraction channels, we set the grating period *P* = 8.358 μm and normal beam incidence. At the operation frequency *f*_0_ = 45.89 THz (*λ*_0_ = 6.533 μm), the three diffraction angles are then defined as |*θ*_−1_| = |*θ*_1_| = arcsin(*λ*_0_/*P*) for the ±1st orders and *θ*_0_ = 0° for the 0th order. To enable active switching of the wavefront in three directions, three target Fermi levels are required in total. Here, we set 0.1 eV and 1 eV as the lowest and highest Fermi levels. For intermediate electrical modulation between these values, a Fermi level of 0.7 eV is chosen, where graphene has an average carrier density of roughly the two cases. The optimized metasurface achieves the absolute efficiencies of 0.311, 0.148 and 0.148 and the relative efficiencies of 0.851, 0.852 and 0.886 for the 0th, −1st and 1st order diffractions respectively as shown in Fig. 5b. We note that efficient gating techniques (e.g., ionic gating) have enabled Fermi level tuning beyond 1 eV, which have demonstrated outstanding performance in applications such as THz light absorption^59,60^. Conventional electrostatic gating across HfO_2_ could also enable access to the graphene Fermi level of 1 eV, considering its high dielectric constant (~25) and dielectric strength (~40 MV/cm)^61,62^. Three-level beam switching metasurface driven by ionic gating, which enables higher graphene carrier density in practical applications, is also theoretically demonstrated in the Supplementary Note 15. We anticipate that, with wider design space and more elaborate optimization methodologies, it should be possible to achieve multi-level single-gate beam switching for more than three levels.

We also note that the required gate voltage for the device operation can be significantly reduced by increasing the device capacitance. This can be achieved by increasing the dielectric constant of the gate dielectric by replacing Al_2_O_3_ with a high-*κ* dielectric such as HfO_2_^61^ and by utilizing lightly doped Si as a mid-IR transparent electrode material^63^. Silicon with a doping concentration under 10^18^ cm^−3^ shows negligible free carrier absorption in mid-IR while remaining sufficiently conductive to function as a gate electrode^64^. Replacing silicon nitride with lightly doped Si in the current structure would secure the optical path length required for optical operation while significantly reducing the effective thickness of the gate dielectric.

Improved device fabrication processes, such as extreme ultraviolet lithography, which enable finer feature sizes with higher precision, can further push the boundaries of the functionalities of this metasurface. Furthermore, the development of charge injection mechanisms to increase the Fermi level of the graphene or the use of alternative two-dimensional materials can lead to higher optical efficiencies. The operation frequency of this study, the mid-infrared, offers substantial advantages for LiDAR and optical communication. The atmospheric window (8–12 μm) exists in this region, and the atmospheric loss rate is significantly lower even under severe weather conditions, compared to the conventionally widely used near-infrared^65^. Thus, it allows for longer detection ranges with lower power consumption regardless of weather conditions, and is safer for human eyes as well as camera sensors. Although this study focuses on the mid-infrared region, the design and analysis principles can be extended to visible and near-infrared frequencies, which are widely adopted for conventional applications.

In conclusion, our work presents an experimental demonstration and comprehensive analysis of the single-gate electro-optic beam switching graphene metasurface, which exhibits a wide deflection angle, high relative efficiency, and uniform absolute efficiency across the diffraction orders originating from Fermi level-dependent interference between a resonant QNM and the background modes. Due to the strong interaction between the constituting optical elements, it is difficult to design such a device with a unit-cell design approach relying upon LPA. Instead, we adopt structural optimization approaches based on a genetic algorithm, providing further advancements in the design of active beam switching metasurfaces. This emerging technology has a lot of potential applications, such as LiDAR, optical communication, a freespace tunable beam splitter, a freespace tunable beam demultiplexer, active directional propulsion for solar sails, beam routing for folded optics, and optical computing. This emerging technology has a lot of potential applications, such as LiDAR, optical communication, a freespace tunable beam splitter, a freespace tunable beam demultiplexer, active directional propulsion for solar sails, beam routing for folded optics, and optical computing The performance of these metasurfaces is expected to improve with further advancements in material science and device fabrication techniques, leading to even more influential applications and discoveries.

## Methods

### Device fabrication

The metasurfaces were fabricated on a 200 nm-thick low-stress silicon nitride membrane (NX10100D, Norcada). A 70 nm-thick gold back reflector with a 3 nm-thick titanium adhesion layer was deposited on the backside of the membrane using thermal evaporation. On the opposite side of the gold back reflector, a 30 nm-thick aluminum oxide layer was deposited via ALD. The top electrode lines were patterned using photolithography with a negative mask and a mask aligner (MJB4 Mask Aligner, SUSS MicroTec), and deposited using thermal evaporation (7 nm titanium/70 nm gold). A CVD-grown graphene was directly wet-transferred from the polymethyl methacrylate (PMMA)/Graphene/polymer layers, which are purchased from Graphenea Inc. The optimized metasurface structure was then patterned using electron beam lithography with a PMMA resist. The entire size of the metasurface was 818 × 377 μm, which is sufficiently larger than the beam spot size. Utilizing the patterned PMMA layer as a soft etch mask, part of the graphene exposed to the air was etched by oxygen plasma asher. The metallic gratings were then formed by lift-off of a 64 nm-thick gold layer with a 7 nm titanium adhesion layer deposited by thermal evaporation. The flow chart of the device fabrication steps is provided in the Supplementary Note 9.

### Measurement

A tunable quantum cascade laser (MIRcat-2400, Daylight Solutions) was employed as a continuous wave monochromatic light source operating in the 6–11 μm frequency range. An infrared step attenuator (102-C, LASNIX) was placed in front of the quantum cascade laser to address the intensity of the laser without distortion of the Gaussian wavefront. The intensity of the light source incident on the metasurface was measured to be 3.37 mW at the operation frequency 41.17 THz. The laser polarization was adjusted to TM mode using two linear polarizers. A gold parabolic mirror focused the laser light to achieve a beam spot diameter of 213 μm at the metasurface. The focused light was incident on the metasurface at an angle of 45° relative to the surface normal vector of the metasurface. The power of the reflected light was measured by a power meter (PM16-401, Thorlabs) mounted on a plate attached to a high-precision rotation stage that rotates around the sample position. The power meter has inherent background signal fluctuations over time. The dark state intensities were measured right before and after the metasurface measurement to linearly compensate for this fluctuation (see Supplementary Note 10). The detector size of the power meter is 10 mm, which can collect the reflected light spreaded over a 7.125° angular region. A Keithley 2400 Sourcemeter was employed to apply the DC gate bias to the metal-dielectric-graphene capacitor and to measure the gate-source and source-drain currents. All measurement was performed in a nitrogen atmosphere to prevent the graphene degradation caused by laser exposure (see Supplementary Note 11).

### Electromagnetic simulation for structure optimization and analysis

The device structure parameters were optimized using a genetic algorithm implemented in MATLAB. The figure of merit of the individual device structure was calculated using RETICOLO V9, an open MATLAB library for RCWA^43,44^. The detailed definition of the figure of merit, design parameters, and the optimization flow chart are described in the Supplementary Note 5. The reflection coefficient in the complex frequency plane was calculated using S4, an open Python library for RCWA^66^. For QNM expansion (Fig. 4b), RPExpand, an open MATLAB library for Riesz projection expansion of resonance phenomena^55^, was combined with S4. To decompose E(r*, f*_0_) and H(r*, f*_0_) of the resonant QNM and the non-resonant background response, each field profile was integrated along the contour surrounding the pole and the operation frequency *f*_0_ using Cauchy’s residue theorem^54^. Details on the integration contour, expansion range, and other simulation settings used to draw Fig. 4 are provided in the Supplementary Note 8. In all simulations and optimization processes, the surface conductivity of graphene was calculated based on the random phase approximation^36^. The refractive index of gold was measured through mid-infrared spectroscopic ellipsometry. For titanium and aluminum oxide, data were obtained from Rakić^67^ and Kischkat^68^, respectively. For low-stress silicon nitride, for which the mid-infrared ellipsometry was not compatible due to its smaller membrane area (1 × 1 mm) than the beam size of the ellipsometer, the reflection spectrum was measured, and its dispersive relative permittivity was fitted using the Brendel-Bormann model to reconstruct the refractive index. The relative permittivity of all materials was analytically continued for the complex frequency analysis (Supplementary Note 12).

## Supplementary information

Supplementary Information

## Data Availability

The authors declare that the data supporting the findings of this study are available within the article and its Supplementary Information files.
