# Supplementary material for: Single-gate electro-optic beam switching metasurfaces
Source: Light Sci Appl. 2025 Aug 27;14:292. doi: 10.1038/s41377-025-01967-y (PMC12381093; doi:10.1038/s41377-025-01967-y)
Supplement: Supplementary file 1 — Supplementary Information [file 41377_2025_1967_MOESM1_ESM.pdf]

Supplementary Information for

Single-gate electro-optic beam switching metasurfaces

Sangjun Han[1,2,†], Jinseok Kong[1,†], Junho Choi[3,4], Won Chegal[3], and Min Seok Jang[1,*]

[*] jang.minseok@kaist.ac.kr

[†] Equally contributed authors

[1] School of Electrical Engineering, Korea Advanced Institute of Science and Technology, Daejeon 34141, Republic of Korea

[2] Department of Mechanical Engineering, University of California, Berkeley, Berkeley, California, 94720, USA

[3] Strategic Technology Research Institute, Korea Research Institute of Standards and Science, Daejeon 34113, Republic of Korea

[4] Department of Physics, Kyung Hee University, Seoul 02447, Republic of Korea

This file includes:

Supplementary Notes 1 to 16

1. Absolute efficiency, relative efficiency and SMSR
2. Electrical properties of the device
3. Metasurface design parameter optimization
4. Design parameter tolerance
5. Spatial inhomogeneity within the metasurface
6. Analysis using locally periodic approximation
7. Poles, zeros, and Riesz projection
8. Optimization of a metasurface with relaxed constraints
9. Device fabrication steps
10. Reduction of the background signal fluctuation
11. Effects of a nitrogen atmosphere
12. Material refractive index fitting
13. Angular divergence of the reflected beam
14. Simulated analysis on the operating bandwidth
15. Three-level beam switching metasurface with ionic gating
16. Possible applications of the platform

Supplementary Note 1. Absolute efficiency, relative efficiency and SMSR

The main performance metrics used in this study are the absolute efficiency, relative efficiency, and side mode suppression ratio (SMSR). The main variables of the metrics are the measurement angle θ and the applied gate bias V_G. The measurement angle θ is defined as an angle between the normal vector of the metasurface and the vector from the center of the metasurface to the center of the powermeter. The absolute efficiency $\eta_{abs}(\theta, V_G)$ is defined as shown in Eq. S1 below:

$$\eta_{\text{abs}}(\theta, V_{\text{G}}) = \frac{P_{\text{MS}}(\theta, V_{\text{G}})}{P_{\text{inc}}}. \tag{S1}$$

Where $P_{MS}(\theta, V_G)$ is the integrated light power reflected from the metasurface within the angular range of (θ - $\Delta\theta/2$, $\theta + \Delta\theta/2$) at the gate bias V_G, and P_{inc} is the incident power. Here, the angular field-of-view of the powermeter $\Delta\theta$ is $\Delta\theta$ = 7.125°. By definition, the absolute efficiency can have a value between 0 and 1. The relative efficiency $\eta_{rel}(\theta, V_G)$ is the performance metric which evaluates how well the beam is concentrated in a single direction. It is defined as shown in Eq. S2 below:

$$\eta_{\text{rel}}(\theta, V_{\text{G}}) = \frac{\eta_{\text{abs}}(\theta, V_{\text{G}})}{\sum_{\theta} \eta_{\text{abs}}(\theta, V_{\text{G}})}. \tag{S2}$$

In this paper, the relative efficiency is defined as the absolute efficiency at an angle θ to the sum of the absolute efficiencies measured from all measurable angles. By definition, the relative efficiency can have a value between 0 and 1. SMSR(V_G), a metric similar to the relative efficiency and already widely used, is defined as shown in Eq. S3 below:

$$\text{SMSR}(V_{\text{G}}) = 10 \log_{10} \frac{\eta_{\text{abs}}\left(\theta_{\text{max,side}}, V_{\text{G}}\right)}{\eta_{\text{abs}}\left(\theta_{\text{max,main}}, V_{\text{G}}\right)}. \tag{S3}$$

Here, $\theta_{max,main}$ and $\theta_{max,side}$ are the angles at which the absolute efficiency maximizes in the main and side lobes, respectively. The SMSR can theoretically reach -∞ dB when the side lobes are perfectly suppressed.

Supplementary Note 2. Electrical properties of the device

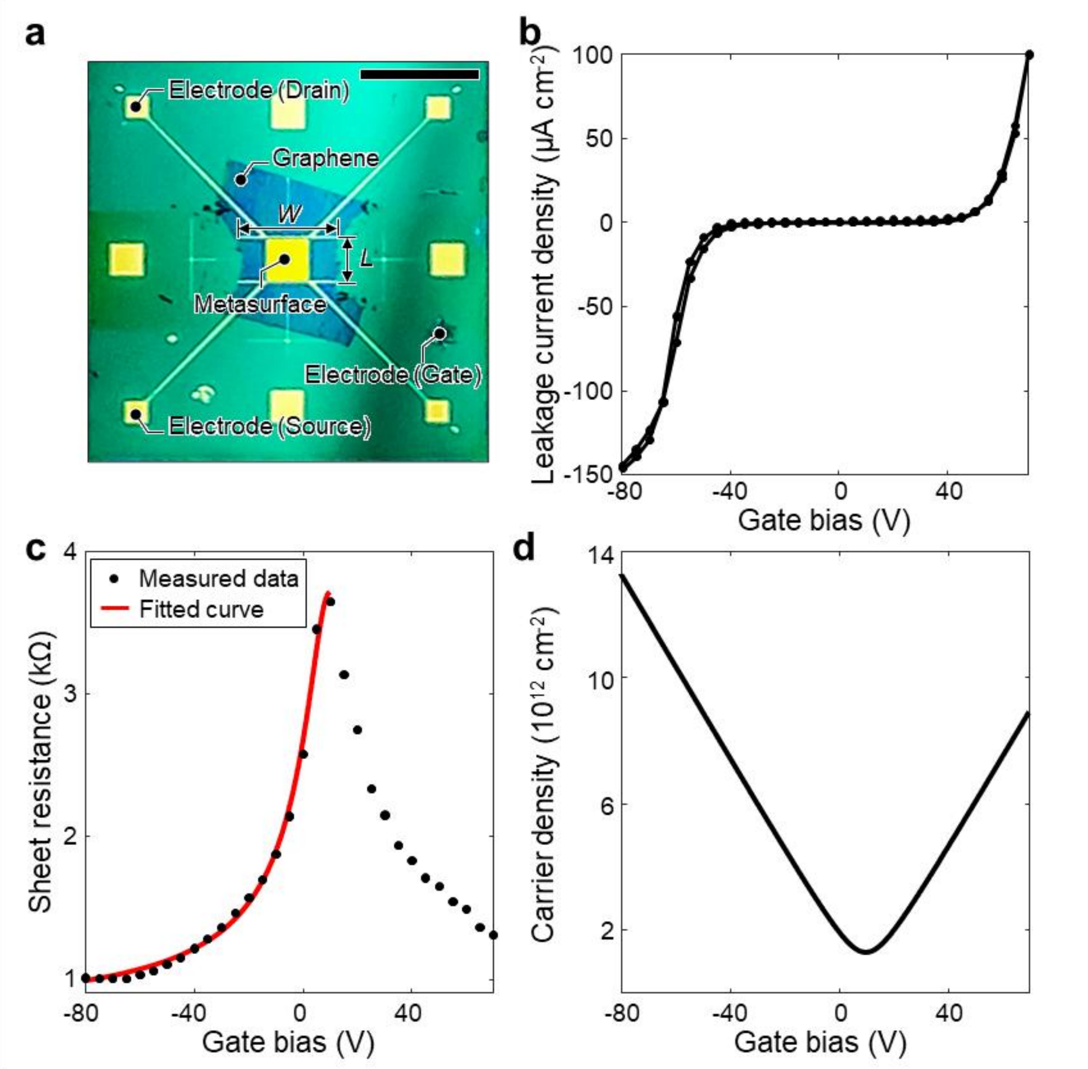

Fig. S1. Electrical properties of the electro-optic beam switching metasurfaces. a Top view image of the sample chip. The scale bar is 3 mm. Gate bias V_G dependent **b** leakage gate-source current density, **c** graphene sheet resistance, and **d** calculated carrier density in the graphene. Here, the gate bias at the CNP of this graphene V_{CNP} is 10 V.

2.1 Leakage current of the dielectric substrate

To measure the gate-source, source-drain current on this metasurface, electrodes were placed around the metasurface as shown in Fig. S1a. The gate electrode is electrically connected to the back reflector, which is the bottom electrode. The source and drain electrodes are located on the front side of the sample chip and have a mirror symmetric structure. These two electrodes are connected by graphene to form a channel with channel length L = 1.0 mm and channel width W = 2.5 mm. The top electrode formed by the graphene and the metasurface, the bottom electrode (a back reflector), and the dielectric substrate between them form a metal-dielectric-graphene capacitor.

The dielectric substrate is 200 nm of low-stress silicon nitride (SiN_x) and 30 nm of aluminum oxide (Al_2O_3). SiN_x exhibits a significant leakage current at high gate bias V_G and is prone to breakdown, limiting the usable Fermi level range of the device. Depositing 30 nm of Al_2O_3 onto SiN_x by atomic layer deposition mitigates this leakage current[1,2]. Fig. S1b shows that this dielectric substrate reliably endures gate bias swing cycles from V_G = -80 V to V_G = 70 V without breakdown and presents negligible hysteresis, suggesting a low density of trap sites in the dielectric substrate.

2.2 Calculation of the doped carrier density in the graphene

From the graphene channel formed between the source and drain electrodes, the carrier density doped in the graphene can be deduced. Figure S1c shows the measured DC sheet resistance as a function of gate bias V_G. The DC sheet resistance R_{tot} is described by Eq. S4 below:

$$R_{\mathrm{tot}} = R_{\mathrm{contact}} + R_{\mathrm{channel}} = R_{\mathrm{contact}} + \frac{L/W}{n_{\mathrm{tot}} e \mu}. \quad \text{(S4)}$$

Here, $R_{contact}$ is the contact resistance between the electrode and graphene, μ is the carrier mobility and n_{tot} is the carrier density in the graphene channel region. The n_{tot} is expressed by Eq. S5 below:

$$n_{\mathrm{tot}} = \sqrt{n_0^2 + \left(\frac{C|V_{\mathrm{CNP}} - V_{\mathrm{G}}|}{e}\right)^2}. \quad \text{(S5)}$$

C is the capacitance of the metal-dielectric-graphene capacitor. n_0 is the residual charge density present even in the charge neutrality point (CNP)[3]. By combining Eq. S4, S5 and fitting the data in Fig. S1c (black dots), the values of $R_{contact}$, n_0 and μ can be determined (red solid line). Figure S1d shows n_{tot} as a function of gate bias V_G. Due to the residual charge density $n_0 = 1.263\times10^{12}$ cm^{-2}, n_{tot} exhibits a larger carrier density than zero even at the CNP (V_G = 10 V). Because of this residual charge density, the experimental value measured at the CNP may present discrepancy with the electromagnetic simulation which calculates Fermi level of graphene at the CNP as 0 eV.

Supplementary Note 3. Metasurface design parameter optimization

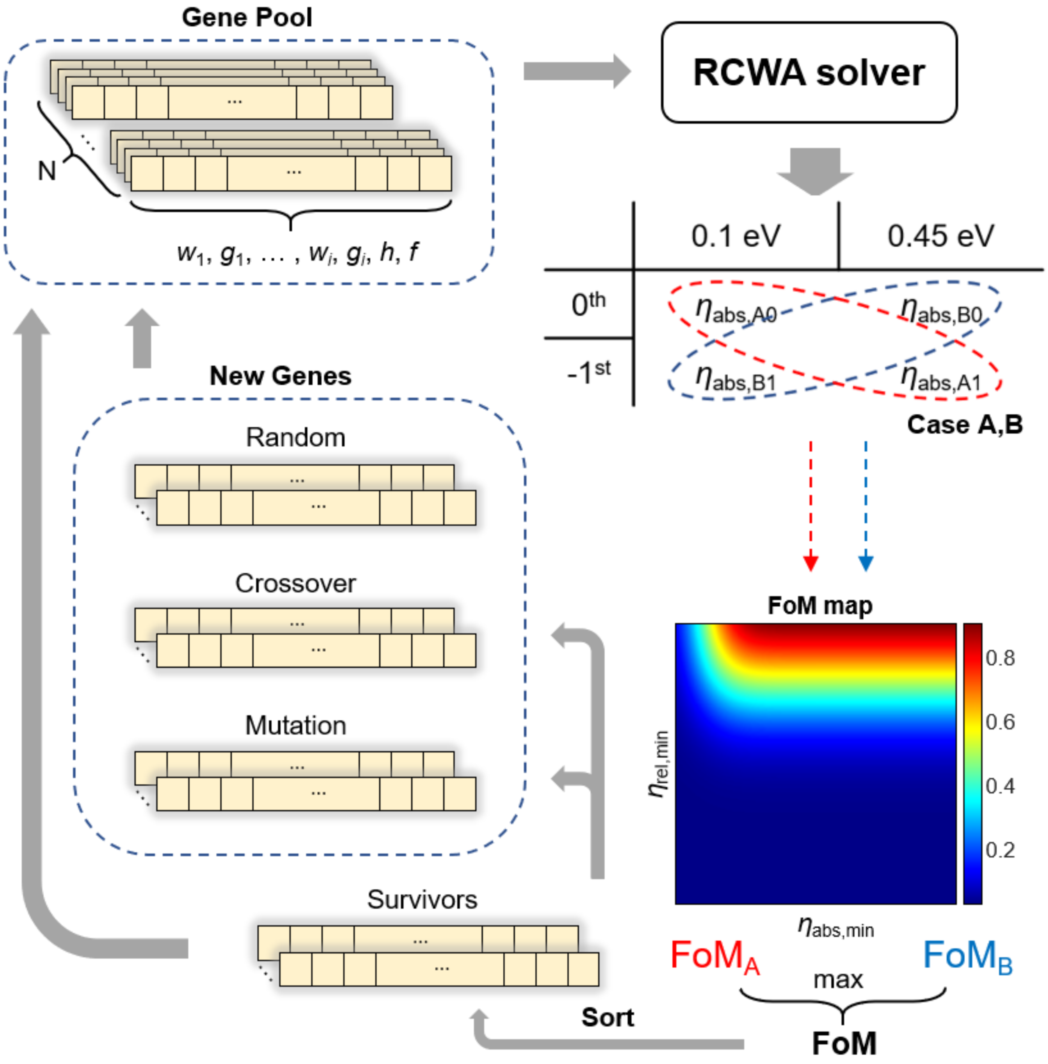

Fig. S2. An overview of the optimization flowchart explaining the implementation of the genetic algorithm and the definition of the applied figure of merit (FoM). Case A is indicated in red and case B is indicated in blue.

3.1 Parameter setting

The device used in this study was optimized according to the process outlined in Fig. S2. The metasurface is designed with five subunits ($1 \leq i \leq 5$) per period, where each subunit consists of a width and a gap. Therefore, the optimization parameters of the metasurface consist of 12 variables, including five widths (w_i), five gaps (g_i), the gold strip height (h), and the operation frequency (f_0). To ensure good adhesion of the gold strip to the dielectric layer, Ti adhesion layer is applied, which has the thickness of $0.1h$, determined by the gold strip height. The lower and upper bounds for each optimization parameter are as follows: 400 nm $\leq w_i \leq$ 3000 nm, 100 nm $\leq g_i \leq$ 1200 nm, 20 nm $\leq h \leq$ 70 nm, 30 THz $\leq f_0 \leq$ 50 THz. The optimization parameters are constrained not only by these

bounds, but also by the limitations of the optical setup, which corresponds to the minimum detectable angle $\theta_{\min}$ = -25°, and the requirement to suppress higher diffraction orders except for the 0th and -1st orders. For a metasurface with period P and incident light at an angle θ_{inc} = 45° with wavelength $\lambda_0 = c_0/f_0$, where c_0 is the speed of light in vacuum, these constraints are expressed as in Eq. S6, S7 below:

$$\sin\theta_{\min} < \sin\theta_{\text{inc}} - \frac{\lambda_0}{P} < 1, \tag{S6}$$

$$\left|\sin\theta_{\text{inc}} - \frac{2\lambda_0}{P}\right| > 1. \tag{S7}$$

The carrier mobility of the graphene is assumed to be 500 $cm^2V^{-1}s^{-1}$.

3.2 Calculation of FoM

The metasurface used in this study operates based on the working principle that the absolute efficiency of each order changes as the Fermi level of graphene transitions between two values. For the optimization, two specific Fermi levels, E_{F1} = 0.1 eV (corresponding to the CNP) and E_{F2} = 0.45 eV, are used. Depending on which diffraction order predominantly occurs at each Fermi level, there are two possible cases, A: 0th order diffraction at E_{F1} and -1st order diffraction at E_{F2}, and B: -1st order diffraction at E_{F1} and 0th order diffraction at E_{F2}. In each case (x = A, B), the figure of merit (FoM_x) is calculated using the minimum absolute efficiency $\eta_{\text{abs,min},x} = \min(\eta_{\text{abs,A}}, \eta_{\text{abs,B}})$ and the minimum relative efficiency $\eta_{\text{rel,min},x} = \min(\eta_{\text{rel,A}}, \eta_{\text{rel,B}})$ of the predominant diffraction orders at the two Fermi levels. The FoM equation is derived by transforming the error function $\text{erf}(x) = \frac{2}{\sqrt{\pi}}\int_0^x e^{-t^2}dt$, which is in the form of step function, as shown in Eq. S8 below:

$$\text{FoM}_x = \left(\text{erf}\left(\left(\eta_{\text{abs,min},x} - 0.1\right) \times 8\right) + 1\right) \times \left(\text{erf}\left(\left(\eta_{\text{rel,min},x} - 0.8\right) \times 4\right) + 1\right)/4. \tag{S8}$$

Here, the values 0.1 and 0.8 represent the target minimum absolute efficiency and the target minimum relative efficiency respectively, as the desired performance for beam switching. The absolute efficiencies are calculated using RETICOLO V9, an open MATLAB RCWA (Rigorous Coupled-Wave Analysis) library[4,5], with a Fourier order of 175. Between the two calculated FoM values, the larger FoM is selected as the final FoM = $\max(\text{FoM}_A, \text{FoM}_B)$.

3.3 Genetic algorithm

The optimization process is conducted using a genetic algorithm, which treats each set of optimization parameters as a "gene"[6,7]. By altering the composition of the gene pool, which is a set of genes, across generations, the genetic algorithm derives the optimal set of parameters. The gene pool consists of four main categories: survivor genes, random genes, crossover genes, and mutation genes. These genes are generated through the following processes.

• Survivor genes: The top 10% of genes from the previous generation, ranked by their FoM, are selected as survivor genes.

• Random genes: Random genes are generated randomly from the parameter space, constrained by the lower and upper bounds of each parameter. The values of each parameter follow a uniform distribution within the parameter space.

• Crossover genes: Crossover genes are generated from three randomly selected survivor genes from the current generation. For each parameter in a crossover gene, a random selection is made from the corresponding parameters of the three parent genes.

• Mutation genes: Mutation genes are generated by selecting one survivor gene from the current generation as a parent. Each parameter of the mutation gene is determined in one of three ways, with specific probabilities assigned to each method. First, a value is sampled from a normal distribution centered on the parent gene's parameter, with a standard deviation of 5%, which is also bounded by the parameter space (50% probability). Second, a value is directly inherited from the parent gene (37.5% probability). Third, a value is randomly sampled from a uniform distribution within the parameter space (12.5% probability).

Only genes generated by the above method that satisfy Eq. S6, S7 are included in the gene pool. Initially, the gene pool starts with 100 random genes. As generations progress, the number of random genes (N_r) gradually decreases and approaches zero while the numbers of crossover genes (N_c) and mutation genes (N_m) increases. By the end of optimization, mutation genes constitute the majority. The detailed changes in the numbers of each type of gene, including the number of survivor genes (N_s), across generations are as follows:

$$\text{generation} = 0: (N_s, N_r, N_c, N_m) = (0, 100, 0, 0),$$

$$1 \leq \text{generation} < 10: (N_s, N_r, N_c, N_m) = (10, 54, 18, 18),$$

$$11 \leq \text{generation} < 20: (N_s, N_r, N_c, N_m) = (10, 0, 45, 45),$$

$$21 \leq \text{generation} < 40: (N_s, N_r, N_c, N_m) = (10, 0, 30, 60),$$

$$41 \leq \text{generation}: (N_s, N_r, N_c, N_m) = (10, 0, 18, 72).$$

After 64 generations, the genetic algorithm ends as FoM converges. The convergence of FoM over generations with the changes in $\eta_{abs,min}$ and $\eta_{rel,min}$ can be observed in Fig. S3a. The final optimal structure derived from the optimization process has gold strip width w_i = (1176, 914, 1491, 1436, 1735) nm, gap g_i = (464, 100, 100, 185,709) nm, gold strip height h = 64 nm, and operation frequency f_0 = 40.49 THz. Theoretically, this structure has FoM of 0.689, exhibiting the absolute efficiency of 0.216 and the relative efficiency of 0.927 in the -1st order diffraction at E_{F1} = 0.1 eV, and the absolute efficiency of 0.217 and the relative efficiency of 0.925 in the 0th order diffraction at E_{F2} = 0.45 eV. The Fourier order convergence of the calculated FoM, $\eta_{abs,min}$, and $\eta_{rel,min}$ for both the optimal structure and the fabricated structure (as presented in Fig. 1) in RCWA simulations can be checked in Fig. S3b, c.

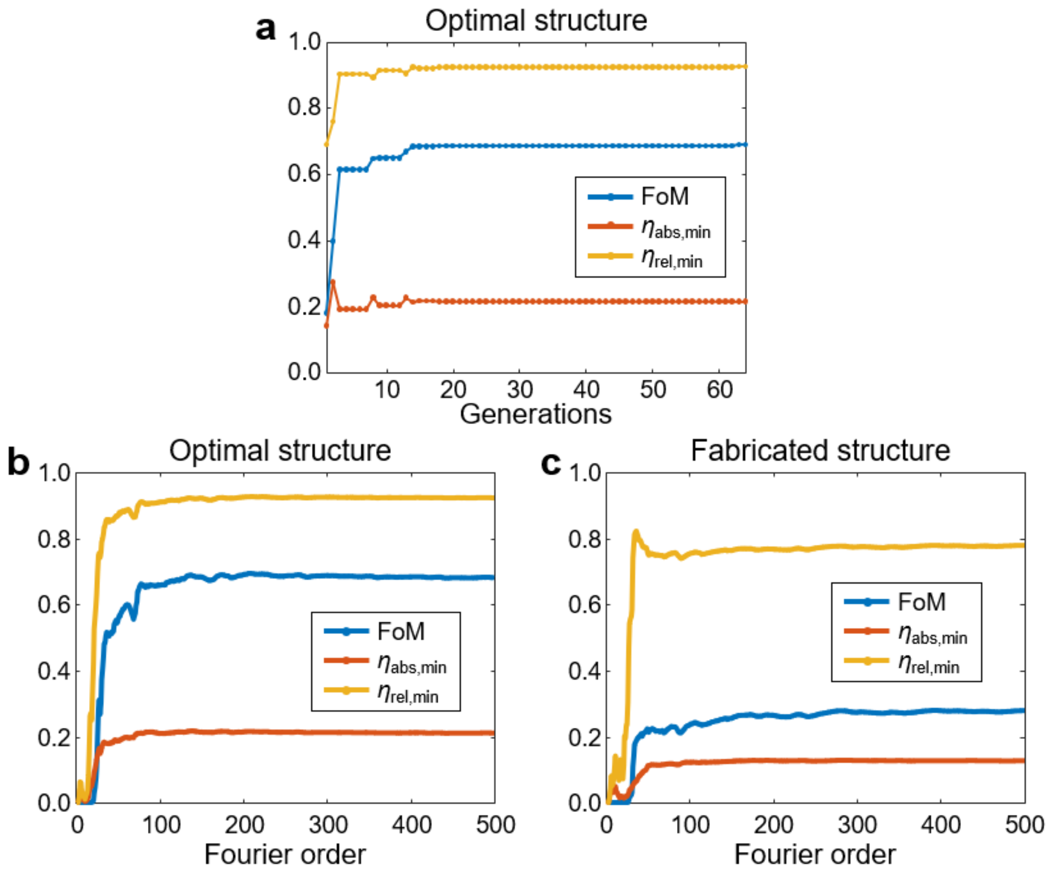

Fig. S3. The convergence of simulation results with increasing generation and Fourier order. a Maximum FoM (blue) and corresponding $\eta_{abs,min}$ (red) and $\eta_{rel,min}$ (yellow) per generation in the genetic algorithm. **b** Fourier order convergence analysis of FoM, $\eta_{abs,min}$, and $\eta_{rel,min}$ for the optimal structure and **c** the fabricated structure.

Supplementary Note 4. Design parameter tolerance

4.1 Optimal structure

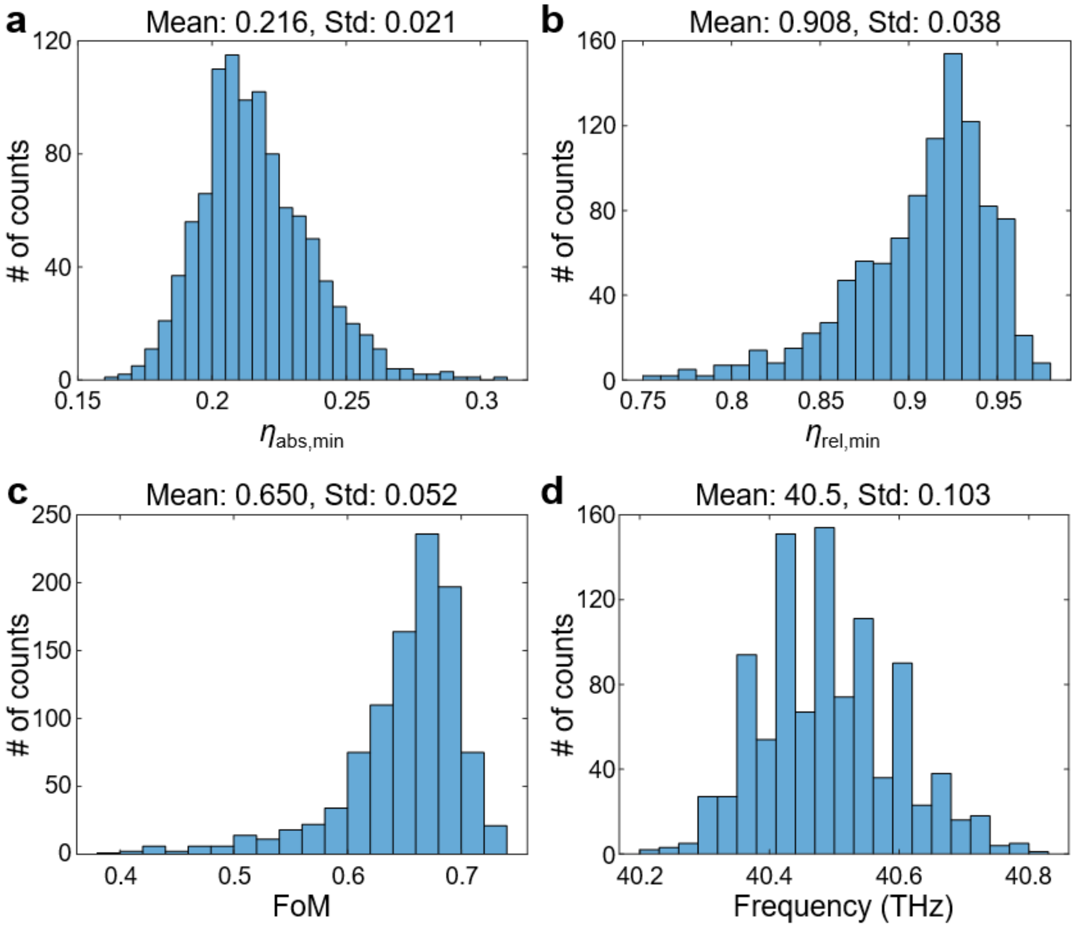

Fig. S4. Results of the Monte Carlo simulation for the optimal structure. a Distribution of $\eta_{abs,min}$ values, with a mean of 0.216 and a standard deviation of 0.021. **b** Distribution of $\eta_{rel,min}$ values, with a mean of 0.908 and a standard deviation of 0.038. **c** Distribution of FoM values, with a mean of 0.650 and a standard deviation of 0.052. **d** Distribution of optimal operation frequencies, with a mean of 40.5 THz and a standard deviation of 0.103 THz.

To evaluate the impact of structural errors from the fabrication process on beam switching performance, the structural tolerance of the optimal structure is assessed using a Monte Carlo simulation[8]. Considering the characteristics of the electron beam lithography (EBL) process, where structural errors primarily occur, fabrication errors (Δ_i) are applied to each subunit by changing the values of w_i and g_i to w_i+Δ_i and g_i-Δ_i respectively, while maintaining the total length of each subunit (w_i + g_i) and gold strip height. Here, Δ_i follows a normal distribution with a mean of 0 nm and a standard deviation of 15 nm. The $\eta_{abs,min}$, $\eta_{rel,min}$, and FoM of the errored structures are

investigated at their optimal operation frequencies, by sweeping the operation frequency within the range of 40.49 THz±1 THz. Figure S4a-d shows the results of the Monte Carlo simulation for 1000 cases. Most of the FoM values of the errored structures are distributed above 0.6, with a mean of 0.650 and a standard deviation of 0.052, which is comparable to the FoM of the optimal structure, 0.689. Additionally, as shown in Fig. S4d, the adjusted operation frequencies are closely distributed around the operation frequency of the optimal structure, 40.49 THz. This theoretical analysis suggests that fabrication errors around 15 nm do not significantly degrade the device's beam switching performance or significantly shift the operation frequency.

In addition to the structural parameters, we examine how the carrier mobility of graphene affects beam switching performance. The mobility tolerance is evaluated by plotting a 2D spectrum of the FoM as a function of operation frequency for carrier mobility values ranging from 10 to 2000 $cm^2V^{-1}s^{-1}$, as shown in Fig. S5. When the carrier mobility is 2000 $cm^2V^{-1}s^{-1}$, a maximum FoM of 0.708 is achieved, which is only marginally higher than the FoM of 0.689 obtained with the carrier mobility of 500 $cm^2V^{-1}s^{-1}$ used in the optimization process. Furthermore, even when the carrier mobility decreases to 150 $cm^2V^{-1}s^{-1}$, the FoM remains at 0.512, indicating that the beam switching performance is not significantly degraded by reduced carrier mobility and is robust to the change of carrier mobility.

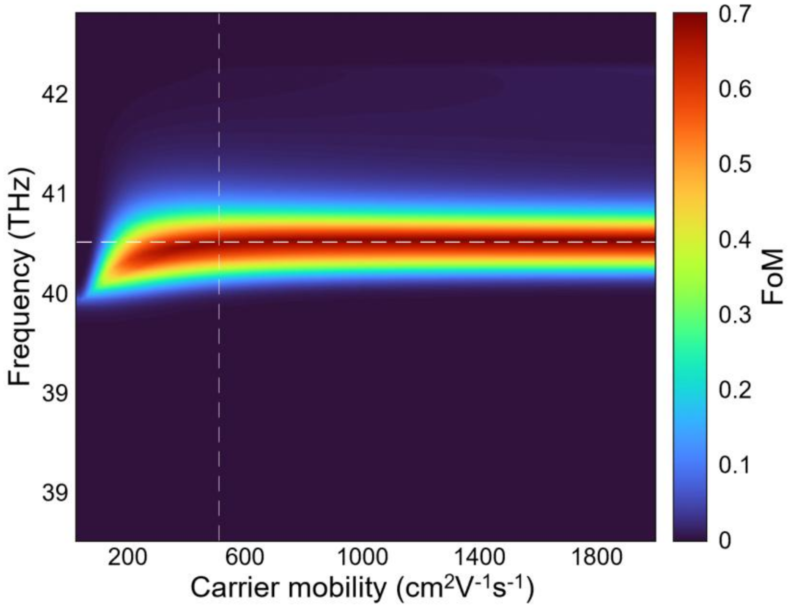

Fig. S5. 2D spectrum of the FoM as a function of operation frequency and graphene carrier mobility for the optimal structure. The location of optimal operation frequency f_0 = 40.49 THz at the carrier mobility of 500 $cm^2V^{-1}s^{-1}$ is indicated by white dashed lines.

4.2 Fabricated structure

The fabricated metasurface does not maintain a uniform pattern across the entire area due to the proximity effect occurring during the EBL process, leading to spatial inhomogeneity[9] (see Supplementary Note 5). As a

result, each structural parameter from different partitions may have a different optimal operation frequency. However, since a single operation frequency is used during measurement, it is important to investigate how spatial inhomogeneity affects the beam switching performance of the fabricated structure. This effect is introduced into the Monte Carlo simulation using the same method applied in the structural tolerance analysis for the optimal structure, except that the operation frequency is fixed to a single value. The Monte Carlo simulation is conducted for 1000 cases with f_0 = 41.17 THz, graphene carrier mobility of 200 cm^{2}V^{-1}s^{-1}, E_{F1} = 0 eV, and E_{F2} = 0.42 eV, based on the experimentally measured absolute efficiency spectra. Fabrication errors with a standard deviation of 15 nm are applied. As shown in Fig. S6, the results show $\eta_{abs,min}$ with a mean of 0.098 and a standard deviation of 0.03, and $\eta_{rel,min}$ with a mean of 0.723 and a standard deviation of 0.123. These values indicate some degradation compared to the simulation result of the fabricated structure without errors, where $\eta_{abs,min}$ = 0.127 and $\eta_{rel,min}$ = 0.773.

Figure S7a illustrates the mobility tolerance of the fabricated structure, evaluated using the same method as for the optimal structure. At a carrier mobility of 200 cm^{2}V^{-1}s^{-1}, FoM of 0.272 is achieved, which is significantly lower than the maximum FoM of 0.639 observed at a carrier mobility of 2000 cm^{2}V^{-1}s^{-1}. The detailed mobility tolerance for the optical efficiencies for each diffraction order at the operation frequency of 41.17 THz is shown in Fig. S7b-e. Unlike when the Fermi level is at the charge neutrality point, a carrier mobility of 200 cm^{2}V^{-1}s^{-1} is insufficient for these values to reach saturation when the Fermi level is 0.42 eV. Precise electron beam patterning with proper proximity effect correction and high-mobility graphene would improve the performance of the device by reducing the discrepancy between design and fabrication.

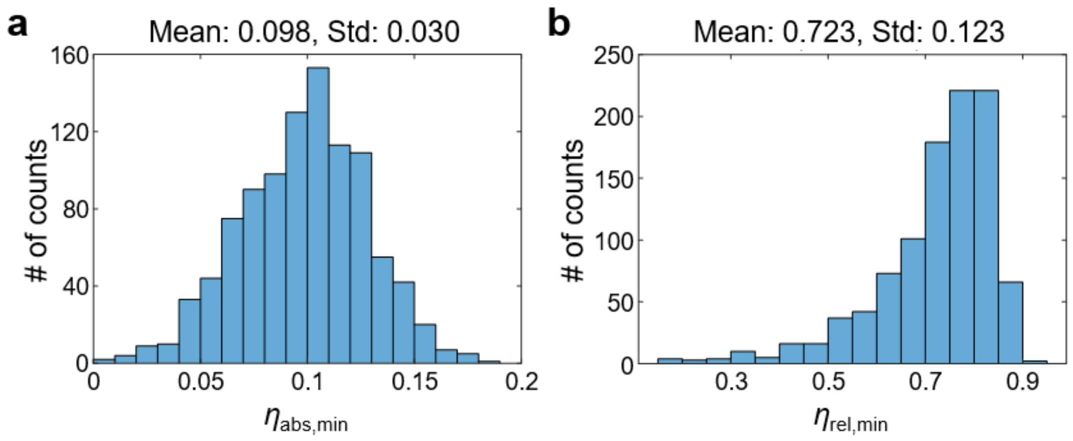

Fig. S6. Results of the Monte Carlo simulation for the fabricated structure. a Distribution of $\eta_{abs,min}$ values, with a mean of 0.098 and a standard deviation of 0.03. **b** Distribution of $\eta_{rel,min}$ values, with a mean of 0.723 and a standard deviation of 0.123.

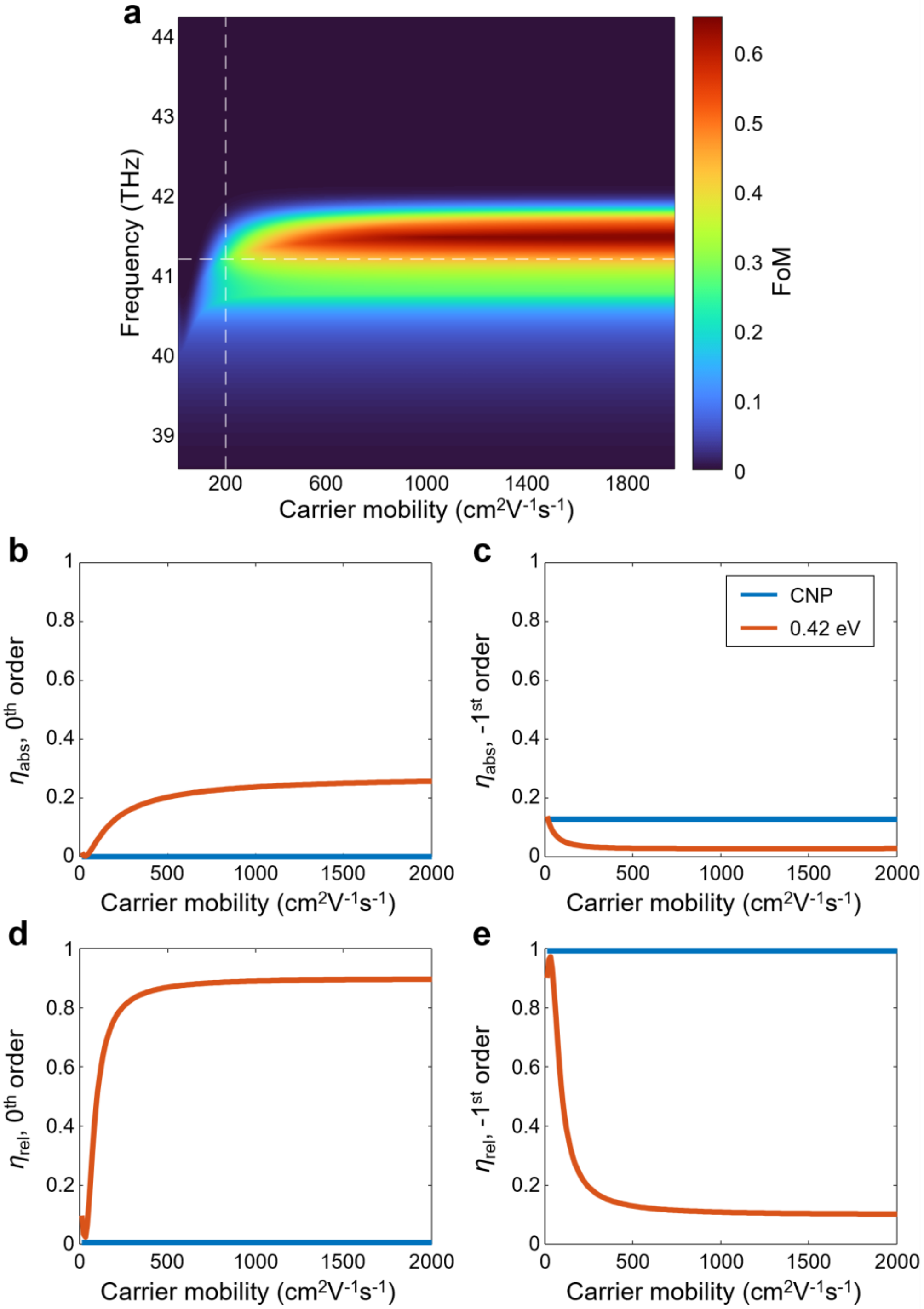

Fig. S7. Carrier mobility tolerance of the fabricated structure. **a** 2D spectrum of the FoM as a function of operation frequency and graphene carrier mobility for the fabricated structure. The location of optimal operation frequency f_0 = 41.17 THz at the carrier mobility of 200 cm^{2}V^{-1}s^{-1} is indicated by white dashed lines. **b** The absolute efficiencies for the 0th and **c** -1st order diffractions, and **d** the relative efficiencies for the 0th and **e** -1st order diffractions of the fabricated structure at f_0 = 41.17 THz as a function of carrier mobility, at the CNP (blue) and E_F = 0.42 eV (red).

These results contrast with the high tolerance observed in the optimal structure, and this discrepancy is related to the incidence angle tolerance. The optimal structure achieves its best performance at an incidence angle of 45°, which is the same value used throughout the simulations. However, as shown in the 2D spectrum in Fig. S8, the optimal incidence angle of the fabricated structure shifts above 45°, indicating that the mobility tolerance analysis of the fabricated structure at a fixed 45° incidence angle may not correspond to that of the optimal structure. Supporting this, the fabricated structure exhibits a high FoM of 0.550 at an incidence angle of 52.5° and an operation frequency of 40.41 THz, even with a low carrier mobility of 200 $cm^2V^{-1}s^{-1}$. This suggests that at its optimal incidence angle, the fabricated structure is likely to exhibit higher mobility tolerance. Due to limitations of the experimental setup, measurements are not performed at incidence angles other than 45°. If measurements could be taken over a wider range of incidence angles, it is anticipated that the device would show better beam switching performance experimentally.

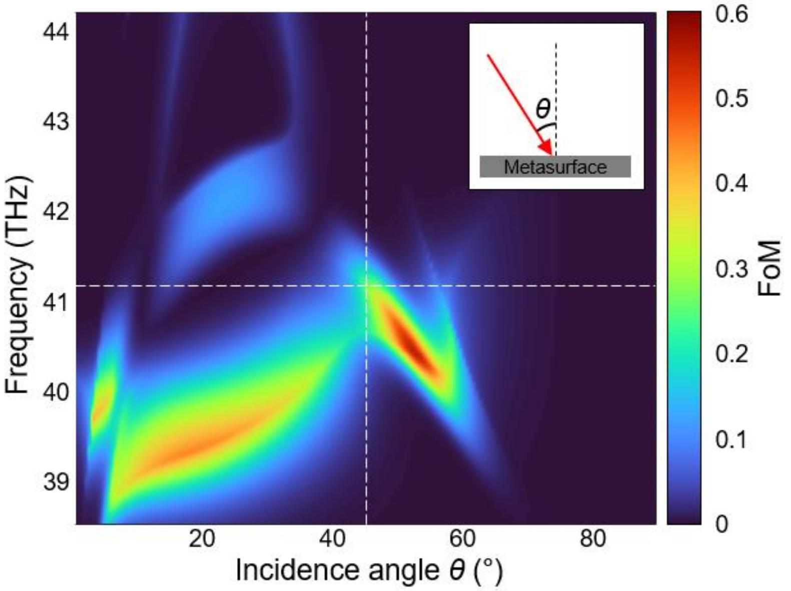

Fig. S8. 2D spectrum of the FoM as a function of operation frequency and incidence angle for the fabricated structure at the carrier mobility of 200 $cm^2V^{-1}s^{-1}$. The location of optimal operation frequency f_0 = 41.17 THz at the incidence angle of 45° is indicated by white dashed lines.

Supplementary Note 5. Spatial inhomogeneity within the metasurface

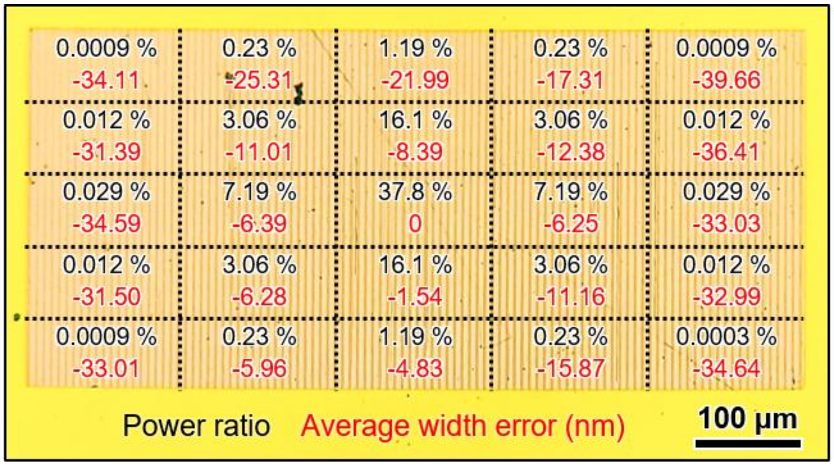

Fig. S9. Spatial inhomogeneity distribution of the incident Gaussian beam power and the structure within the metasurface area.

During the metasurface fabrication, a proximity effect can occur during e-beam lithography over a large area. Due to this proximity effect, at the edges of the metasurface, structures can form with different gold strip widths w_i ($1 \leq i \leq 5$) from the center while the length of the subunit w_i+g_i is the same. Also, since the incident laser is a Gaussian beam, there is a difference in illuminated intensity depending on the position of the metasurface. Fig. S9 presents the spatial inhomogeneity of the incident Gaussian beam power and the structure by dividing the entire metasurface area into 5×5 regions. The average width error Δw_{avg} (red numbers) is defined as the average of the differences between the gold strip width w_{i} with that of the center region $w_{i,\text{center}}$. (i.e. $\Delta w_{\text{avg}} = (\Sigma_{i=1\text{-}5}\,(w_i\text{-}w_{i,\text{center}}))/5$.) The gold strip widths of each region are measured with a scanning electron microscope. The black numbers are the theoretically calculated ratio of Gaussian beam power, that obliquely illuminates the region with 45°, to the total incident Gaussian beam power. Here, the Gaussian beam diameter is 213 μm. 37.8% of the incident Gaussian beam illuminates the center region. Most of the remaining 62.2% of the beam illuminates the regions that exhibit marginal average width errors. In order to reconstruct the optical response of the corresponding metasurface with electromagnetic simulation very accurately, both spatial inhomogeneity of the Gaussian beam and the metasurface structure might need to be considered.

Supplementary Note 6. Analysis using locally periodic approximation

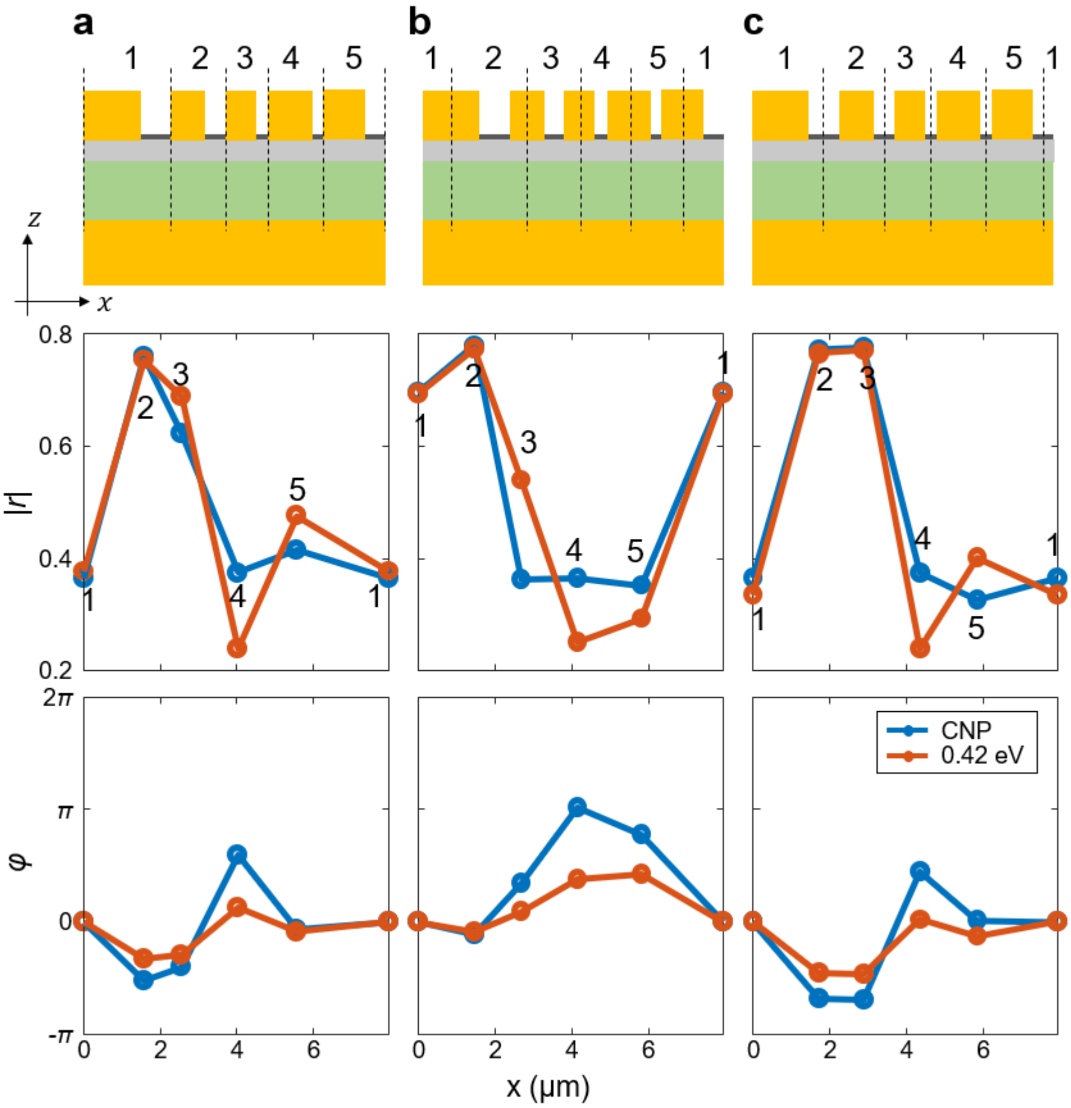

Fig. S10. Analysis using locally periodic approximation. Schematics of divided unit-cells (top panel), spatial reflection amplitude distributions (middle panel), and spatial reflection phase distribution (bottom panel) of metasurface divided in three different ways. Metasurfaces are divided into five unit-cells by vertical lines crossing **a** the left side of the gold strip, **b** the center of the gold strip, or **c** the center of the gap.

We analyze the electro-optic beam switching mechanism using a unit-cell method based on the locally periodic approximation (LPA). We divide this metasurface into five subwavelength unit-cells with different gold strip widths and gaps to get the spatial distribution of the local optical response. There are several ways to divide the metasurface grating into unit-cells. This is illustrated in Fig. S10 (top panel). According to LPA, the local optical response of each unit-cell is assumed to be equal to the optical response of a virtual structure of which the unit-cell is spatially repeated infinitely periodically[10]. Figure S10 shows the spatial distribution of the complex

reflectivity $|r|\exp(i\varphi)$ within one grating period of the metasurface divided by three different methods. The simulations were performed at a frequency of f_0 = 41.17 THz with RETICOLO V9, an open MATLAB RCWA library[4,5]. The electro-optic beam switching behavior of this structure deflected to the -1st order diffraction at the CNP should exhibit a spatially increasing reflection phase distribution from 0 to 2π within one grating period and a uniform reflection amplitude distribution[11], but the bottom panel of Fig. S10 shows that none of these methods reproduce this behavior. This is because there is non-negligible interference between neighboring unit cells, and each cannot be considered in isolated metaatoms. Thus, in order to design or analyze our electro-optic beam switching metasurfaces, we should utilize another method beyond the LPA.

Supplementary Note 7. Poles, zeros, and Riesz projection

7.1 Fermi level dependency of the position of poles and zeros

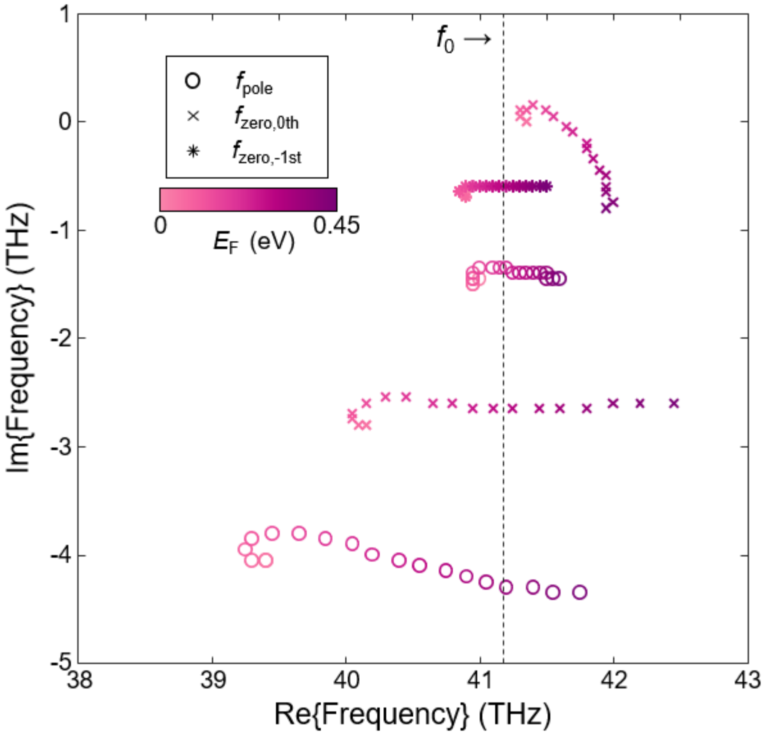

Fig. S11. Movement of the pole and zero positions of this metasurface as the graphene Fermi level changes.

Figure S11 shows movements of the QNM pole and the zeros corresponding to the 0th and -1st order diffraction by the graphene Fermi level $|E_F|$. The behavior of these poles can be explained by perturbation theory[12,13]. The resonance frequency shift $\Delta\omega$ can be approximated to the first-order, leading to:

$$\Delta\omega = -\frac{\omega_0}{2}\frac{\int \Delta\varepsilon(\mathbf{r})|\mathbf{E}(\mathbf{r})|^2 dv}{\int \varepsilon(\mathbf{r})|\mathbf{E}(\mathbf{r})|^2 dv}. \quad \text{(S9)}$$

Where ω_0 and $\mathbf{E}(\mathbf{r})$ are the resonance frequency of the mode and its electric field, respectively. $\varepsilon(\mathbf{r})$ and $\Delta\varepsilon(\mathbf{r})$ are the permittivity distribution of the materials and its variation by the tuning parameter, respectively. For this metasurface, the tuning parameter is the Fermi level E_F of graphene, and $\Delta\varepsilon(\mathbf{r})$ has a non-zero value only at graphene sites. The volume integral is performed over one grating period of the metasurface. Since the metasurface has a more non-local electric field than a typical graphene metasurface[14-16], the electric field $\mathbf{E}(\mathbf{r})$ is weak enough to apply a first-order perturbation approximation in Eq. S9, and the resonant frequency shift $\Delta\omega$ is roughly proportional to the change in graphene permittivity $\Delta\varepsilon(\mathbf{r})$. As $|E_F|$ increases, the QNM pole first redshifts slightly and then blueshifts as $|E_F|$ becomes larger. This is directly related to the change in the real part of the graphene permittivity with $|E_F|$. As $|E_F|$ becomes larger, the graphene becomes more conducting, and the real part of the

graphene permittivity becomes more negative ($\Delta\varepsilon(\mathbf{r}) < 0$). Therefore, according to Eq. S9, resonance frequency $\Delta\omega$ blueshifts for large $|E_F|$. And since the position of zeros are determined by the interference between the QNMs and the background response, they show a similar behavior to the QNM poles.

7.2 Quasinormal mode analysis with extended imaginary frequencies

In addition to the QNM introduced in the main paper, there are other QNMs of this metasurface that are very far from the real operation frequency. If we extend the imaginary frequency in Fig. 4a, there is another QNM_2 below the QNM_1 introduced in the main paper as shown in the Fig. S12a. Figure S12c, d decomposes the complex diffraction coefficients of this metasurface into the contributions of the two resonant QNMs and a 'new' background response. The new background response is the sum of the contributions from QNMs that are further away from the real operation frequency than QNM_2 [17]. QNM_2 is a much more lossy resonance than QNM_1, so its contribution (blue dotted lines) has a very broad resonance lineshape along the real frequency axis. This resonance is almost spectrally uniform, shifting slightly up and down with E_F. The new background response (yellow dotted lines, BG) exhibits a non-resonant and almost independent behavior with respect to E_F. The complex sum of the complex diffraction coefficients of QNM_2 and the new background response, QNM_2+BG, gives the green solid lines which agrees well with the behavior of the black dotted lines in the upper panel of Fig. 4b. The gray line in the Fig. S12d is the phase difference between QNM_1 and the QNM_2+BG, which agrees well with the black solid lines in the lower panel of Fig 4b. This implies that the 'background' response spectrum in Fig. 4b can be decomposed as the sum of the QNM_2 and the new background response spectrum, and the marginal E_F dependence of the 'background' response in Fig. 4b can be attributed to the presence of QNM_2 inherent in it.

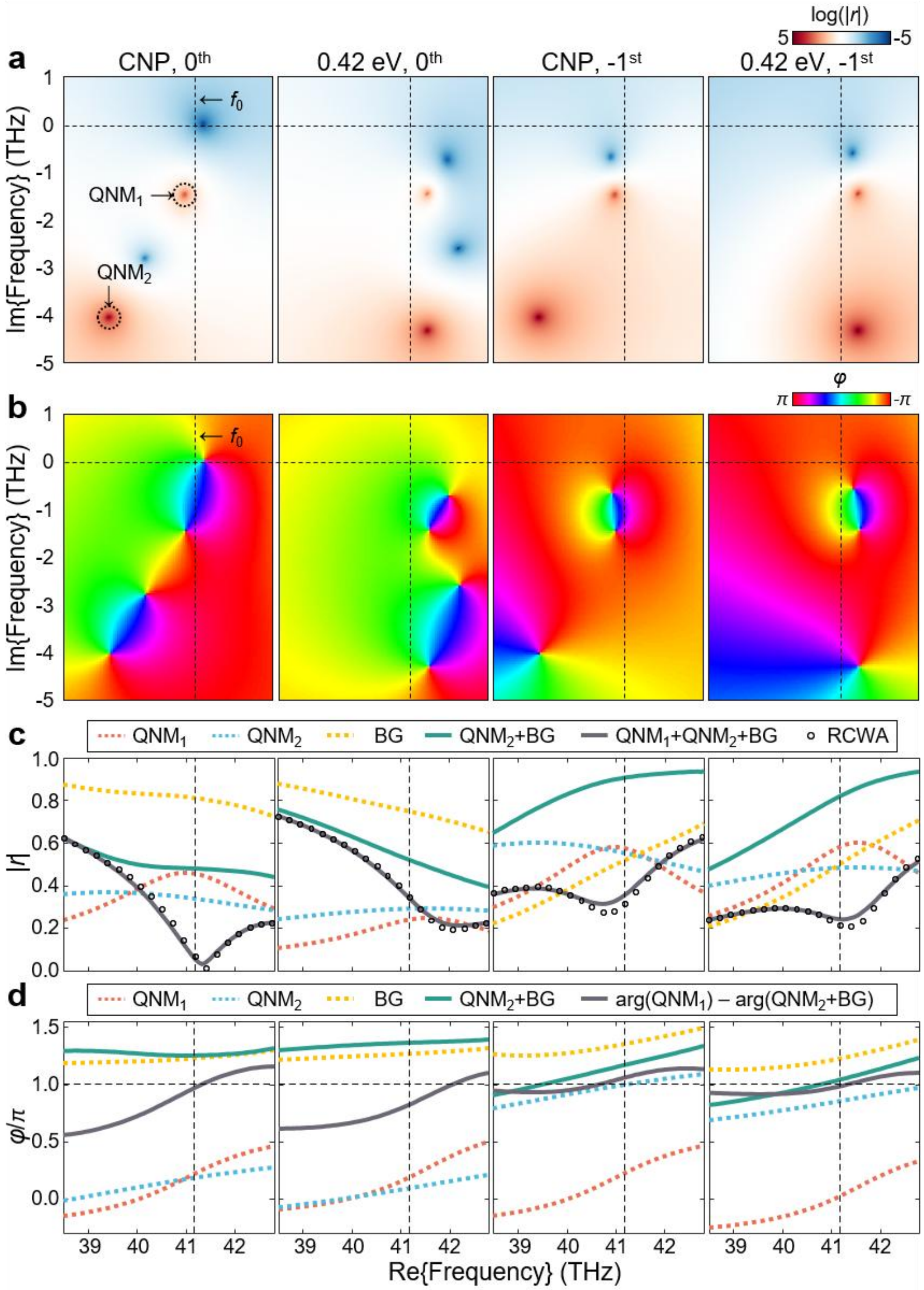

Fig. S12. Quasinormal mode analysis with extended imaginary frequencies. Spectra of the complex diffraction coefficient $|r|\exp(i\varphi)$ with extended imaginary frequencies. **a** Logarithm of the amplitude $\log(|r|)$ and **b** phase φ are depicted for the 0th and -1st order diffraction at the CNP and E_F = 0.42 eV. **c** Amplitude $|r|$ and **d** phase φ/π spectra decomposed with contributions of the QNM_1, QNM_2 and the background response (BG) using Riesz projection (dotted lines). In **c**, reconstructed amplitude spectra (gray solid lines) show good agreement with the electromagnetically simulated spectra (black circles). Green solid lines in **c** and gray solid lines in **d** are auxiliary spectra that exhibit similar behavior with the 'background' response spectra in Fig. 4b. explaining its marginal E_F dependence.

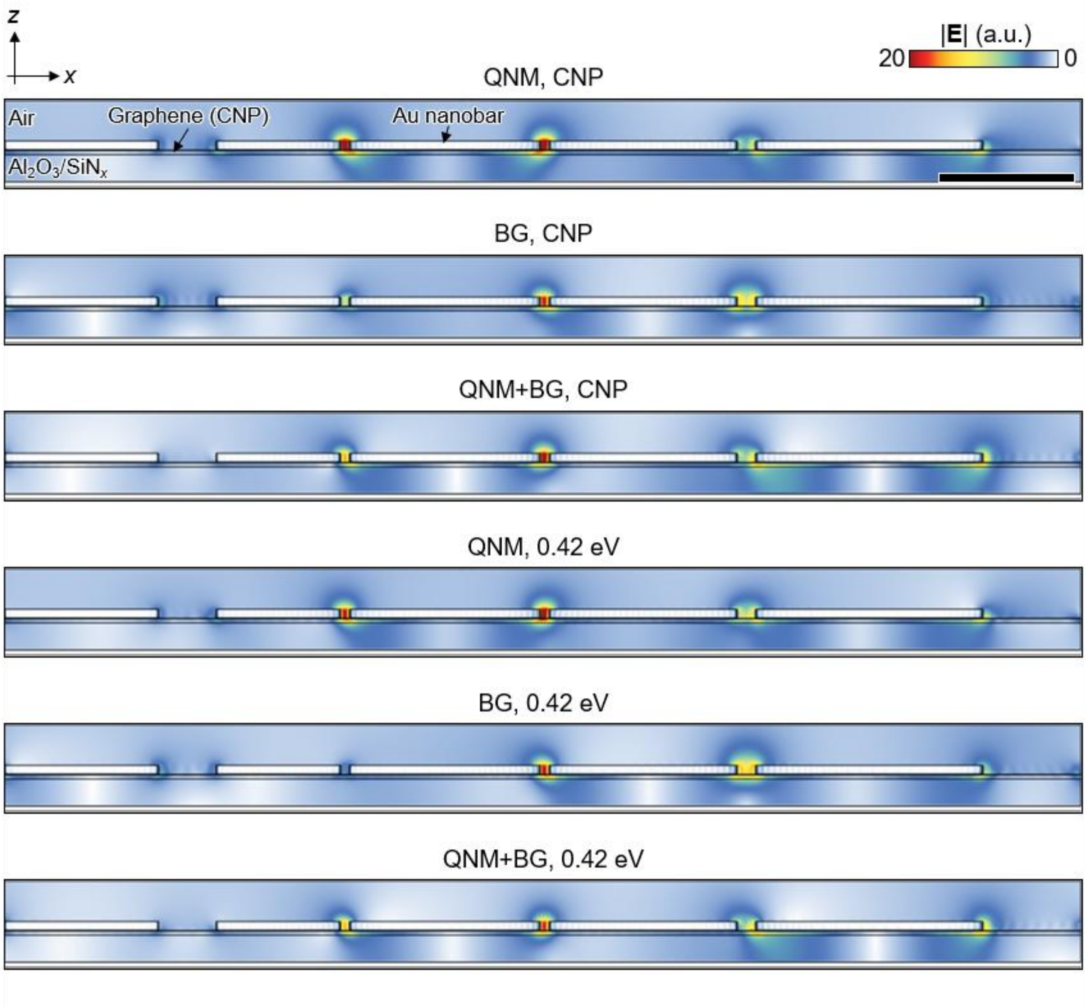

Fig. S13. Decomposed (QNM, BG) and reconstructed (QNM+BG) electric field intensity profiles over one grating period of the metasurface at the CNP and E_F = 0.42 eV. The scale bar is 1 μm.

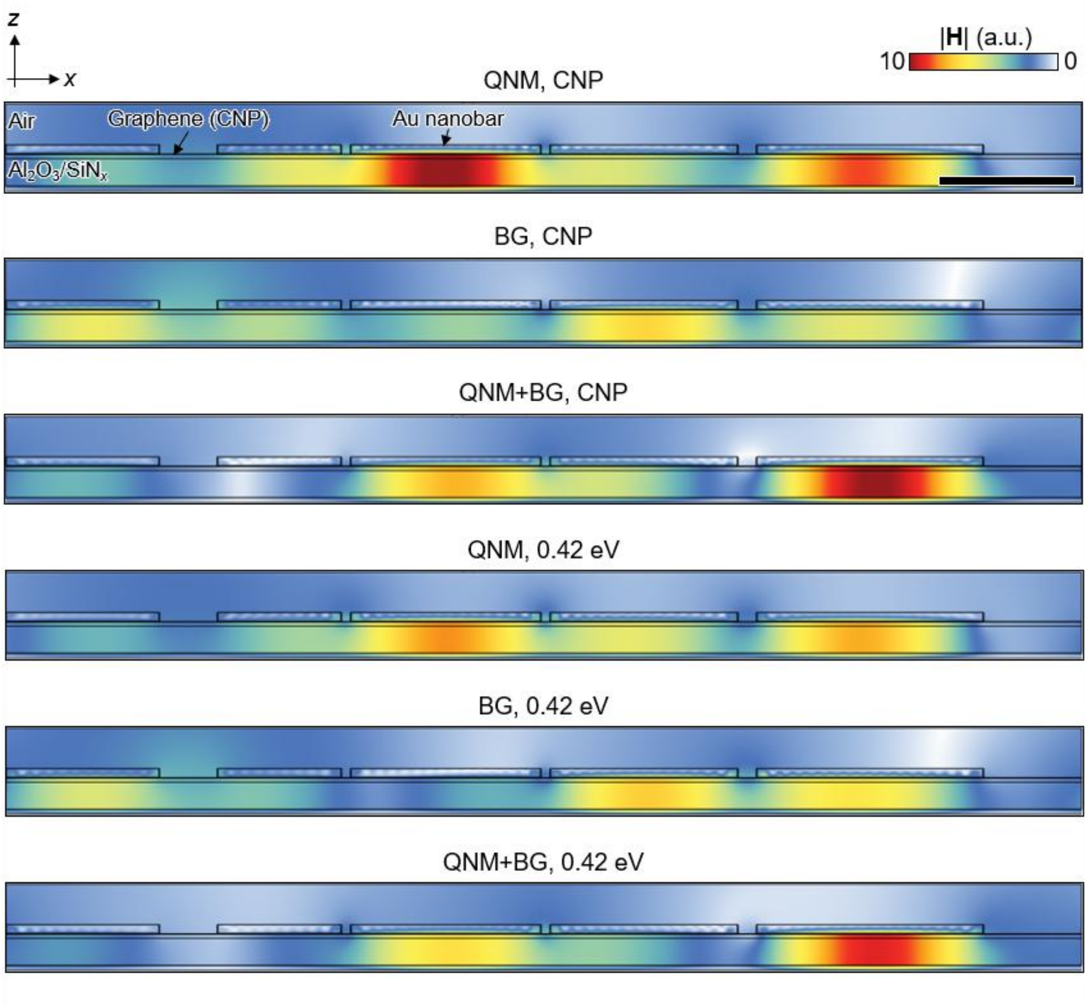

Fig. S14. Decomposed (QNM, BG) and reconstructed (QNM+BG) magnetic field intensity profiles over one grating period of the metasurface at the CNP and E_F = 0.42 eV. The scale bar is 1 μm.

7.3 Riesz projection and contour integrals

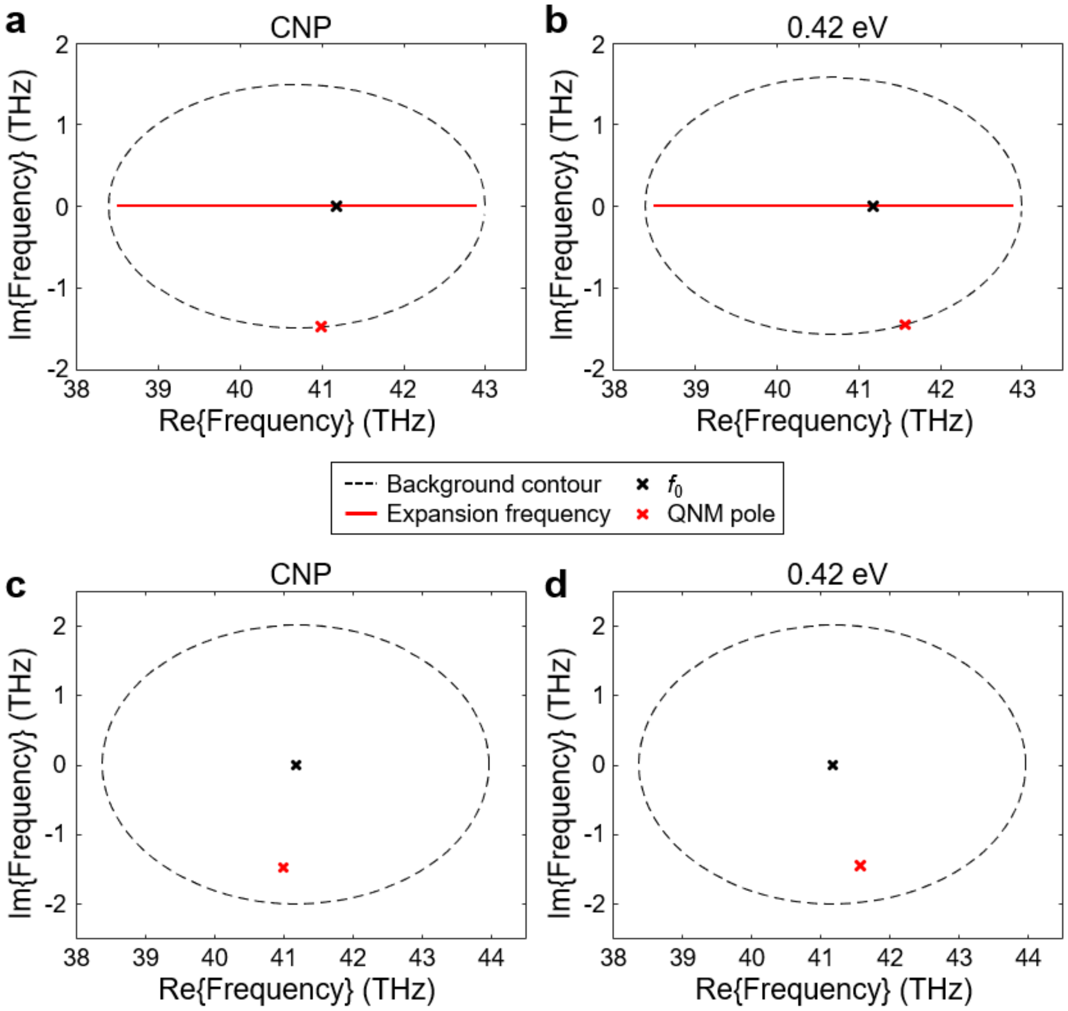

Fig. S15. Cauchy's integral contours. Cauchy's integral contours (C_{BG}, black dashed lines) to perform the Riesz projection (Fig. 4b) at **a** the CNP and **b** E_F = 0.42 eV, and to decompose electromagnetic field profiles (Fig. S13, S14) at **c** the CNP and **d** E_F = 0.42 eV. In **c** and **d**, contour C_k is a circle with a radius of 0.01 THz centered at f_k (red x-marks).

To decompose complex diffraction coefficients or electromagnetic profiles, Riesz projection is performed. We firstly define $q(\omega_0)$ as the analytic continuation of the physical observable of interest. Applying Cauchy's residue theorem to $q(\omega_0)$, we obtain the expansion shown below[17]:

$$q(\omega_0) = \sum_{k=1}^{n} q_k(\omega_0) + q_{\mathrm{BG}}(\omega_0)\,. \tag{S10}$$

Where $q_{BG}(\omega_0)$ corresponds to the contribution of QNMs outside the region of interest, which is the non-resonant background response. The $q_k(\omega_0)$ is the Riesz projection of $q(\omega_0)$, for a particular k-th pole. $q_k(\omega_0)$ and $q_{BG}(\omega_0)$ are defined by the following contour integrals:

$$q_k(\omega_0) = -\frac{1}{2\pi i}\oint_{C_k} \frac{q(\omega)}{\omega-\omega_0}d\omega = \frac{1}{2\pi i}\oint_{C_{BG}} \frac{q(\omega)}{\omega-\omega_0}\prod_{\substack{m=1\\m\neq k}}^{n}\frac{\omega-\omega_m}{\omega_k-\omega_m}d\omega\,, \tag{S11}$$

$$q_{BG}(\omega_0) = \frac{1}{2\pi i}\oint_{C_{BG}} \frac{q(\omega)}{\omega-\omega_0}d\omega\,. \tag{S12}$$

Here, the contour C_{BG} includes both the frequency of interest $\omega_0 = 2\pi f_0$ and the poles $\omega_k = 2\pi f_k$, and the contour C_k includes only a single k-th pole. If the position of the pole is precisely defined, then using the algorithm proposed in Ref. 18, q_k can be computed using only C_{BG} instead of the contour integral over C_k. To apply this algorithm, we utilize RPExpand, an open MATLAB library[17]. To compute the Riesz projection for complex diffraction coefficients, the integration contours C_{BG} are set as shown in Fig. S15a, b. The contours are set to include the real frequency range f = [38.5, 42.9] THz (red solid lines) and the frequency of interest f_0 (black x-mark), as well as the QNM resonance frequency f_k (red x-mark), which varies with E_F. To decompose the 0th and the -1st order diffraction coefficients, the integrands are calculated at 150 and 120 discrete points along the corresponding contour line C_{BG}, respectively. To calculate the electromagnetic field intensity $\mathbf{E}(\mathbf{r}, f_0)$ and $\mathbf{H}(\mathbf{r}, f_0)$ in Fig. S13, S14, C_{BG} is set as shown in Fig. S15c, d. C_k is set as a circle with a radius of 0.01 THz centered at f_k. To decompose the electromagnetic field intensity, integrands are calculated at 150 and 25 discrete points on C_{BG} and C_k, respectively. Here, each integrand value is calculated using S^4, an open Python RCWA library, and the number of the Fourier orders utilized in the RCWA simulation is 175. All material permittivities used in the simulation are analytically continued to the complex frequency as described in the Supplementary Note 12.

Supplementary Note 8. Optimization of a metasurface with relaxed constraints

8.1 2-level beam switching

As relaxed constraints, the graphene carrier mobility is increased from 500 $cm^2V^{-1}s^{-1}$ to 1000 $cm^2V^{-1}s^{-1}$. Additionally, the lower bounds for the gold strip width w_i, and gap g_i are reduced to 20 nm, while the upper bound for the gold strip height h is increased to 100 nm. To account for the reduced minimum feature size, RCWA simulations are performed with a Fourier order of 350. Except for these adjustments, the optimization process follows the same methodology as described in Supplementary Note 3, including the restriction condition in Eq. S6, S7. The final optimal structure is derived with the parameters w_i = (1110, 1449, 22, 1096, 1194) nm, g_i = (483, 312, 386, 26, 20) nm, h = 41 nm, and f_0 = 44.16 THz.

8.2 3-level beam switching

The same relaxed constraints used in the optimization of the 2-level beam switching metasurface are applied. However, for the design of the 3-level beam switching metasurface, which utilizes a 0° incidence angle and the 0th, -1st, and 1st order diffraction channels, the restriction conditions of the parameter set are modified to suppress higher diffraction orders, as described in Eq. S13, S14:

$$\frac{\lambda_0}{P} < 1, \tag{S13}$$

$$\frac{2\lambda_0}{P} > 1. \tag{S14}$$

For RCWA simulations, a Fourier order of 300 is used. The final optimal structure is derived with the parameters w_i = (1503, 1178, 1446, 1972, 538) nm, g_i = (1073, 248, 141, 79, 179) nm, h = 74 nm, and f_0 = 45.89 THz.

Supplementary Note 9. Device fabrication steps

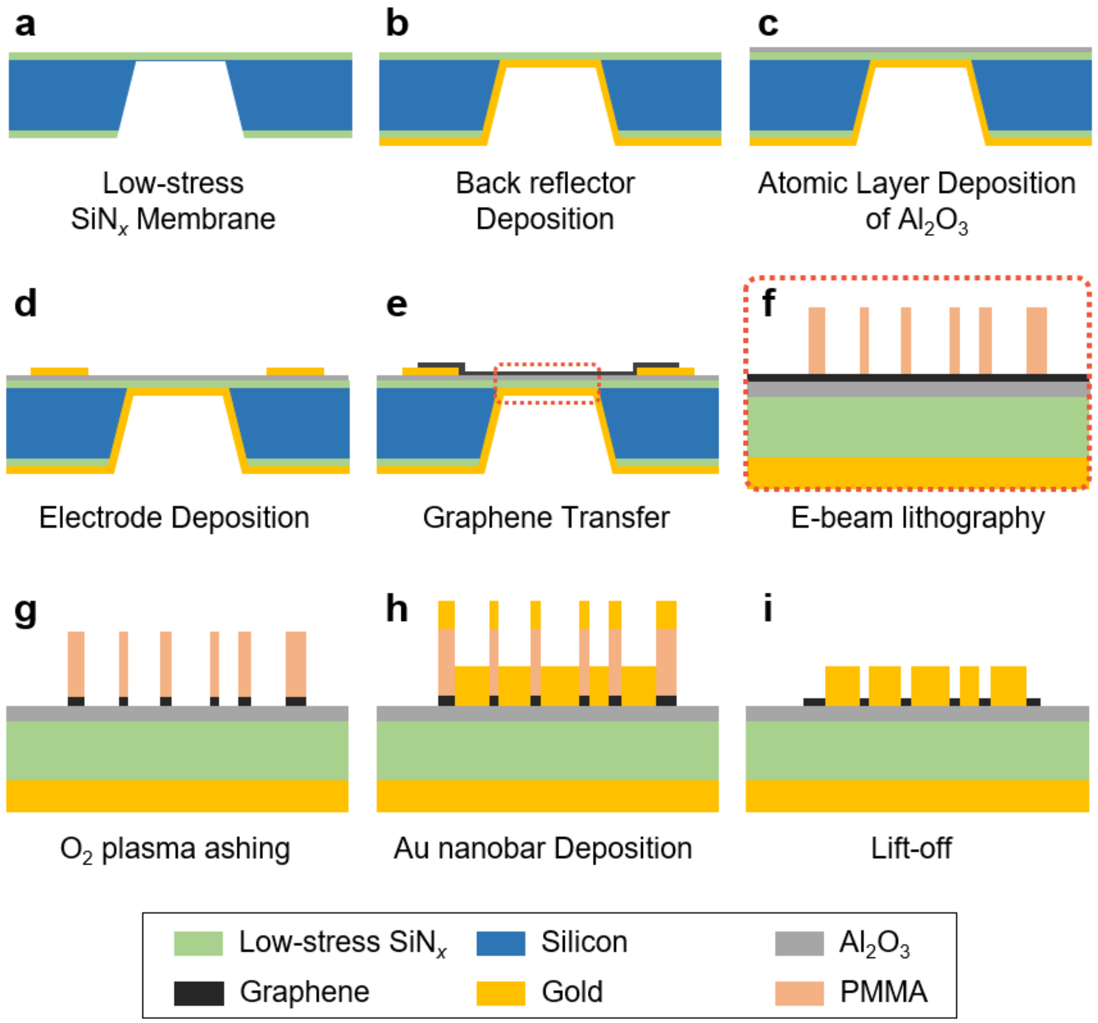

Fig. S16. Steps for fabricating the device. a A 200 nm-thick low-stress silicon nitride membrane supported by a silicon frame is prepared. **b** A 70 nm gold back reflector with a 3 nm Ti adhesion layer is deposited on the backside of the silicon nitride membrane using a thermal evaporator. **c** A 30 nm Al_2O_3 layer is deposited on the front side of the silicon nitride membrane using atomic layer deposition (ALD). **d** Electrode lines consisting of 7 nm of Ti for adhesion and 70 nm of gold are deposited on the front side of the silicon frame using photolithography and a thermal evaporator. **e** The monolayer graphene is wet-transferred to sufficiently cover the electrode lines and the entire membrane. **f** PMMA is deposited on the graphene layer and subsequently patterned on the designated area of the membrane where the metasurface will be formed using e-beam lithography (EBL). **g** The part of the graphene exposed by the patterned PMMA is etched using an O_2 plasma asher. **h** 6 nm of Ti for adhesion and 64 nm of gold are deposited on the exposed Al_2O_3 layer and patterned PMMA using a thermal evaporator. **i** The grating is formed by removing the PMMA and the gold deposited on it using a lift-off process with acetone.

Supplementary Note 10. Reduction of the background signal fluctuation

The powermeter we utilize for our optical measurements (PM16-401, Thorlabs) has an inherent background signal fluctuation. This fluctuation appears linearly on measurement time scales of a few hundred seconds. Measuring the background optical signal in a dark-state with no incident laser light before and after the main measurement, we compensated the raw data with a linear function of time to calculate the time-compensated power $P_{\text{compensated}}$ as shown in Eq. S15 below:

$$P_{\text{compensated}}(t) = P_{\text{raw}}(t) - \left(\frac{P_{\text{dark,end}} - P_{\text{dark,start}}}{t_{\text{end}} - t_{\text{start}}}(t - t_{\text{start}}) + P_{\text{dark,start}}\right). \quad \text{(S15)}$$

Where P_{raw} is the raw power measured at time t. In other words, it is the power value reflected from the metasurface during the gate bias cycle. $P_{\text{dark,start}}$ and $P_{\text{dark,end}}$ are the background power measured in the dark-state before and after the main measurement, respectively. t_{start} and t_{end} are the start and end times of the measurement, respectively.

Supplementary Note 11. Effects of a nitrogen atmosphere

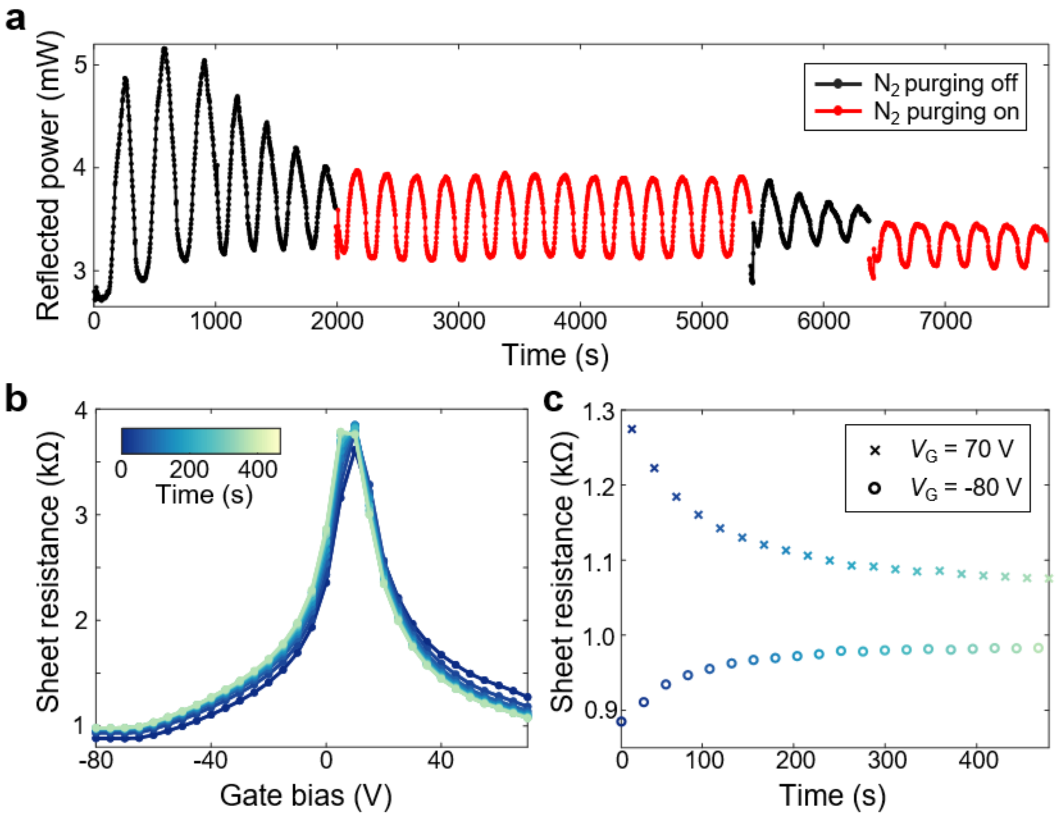

Fig. S17. Effects of a nitrogen atmosphere around metasurfaces. a Modulation cycles of reflected power from the metasurfaces over time. The nitrogen purging is turned on (red lines)/off (black lines) during the cycles. **b** Graphene sheet resistance-gate bias graph over time after nitrogen purging is turned on. V_{CNP} is slightly shifted and saturated over time. **c** The graphene sheet resistivity over time at V_G = 70 V and V_G = -80 V, the two ends of the gate bias sweep cycle.

11.1 Nitrogen purging to prevent the graphene burning

During the measurement, the high power of the focused CW laser can degrade the graphene in real time. This is due to local heating caused by the high power of the laser and the high absorption of the graphene metasurface, which makes the graphene to oxidize and disappear. To remove oxygen molecules around the graphene metasurface, a nitrogen atmosphere is provided around the metasurface. Figure S17a presents the modulation range of the reflected laser power from the metasurface when nitrogen purging is on and off. Several modulation cycles are measured over time while varying the gate bias from V_G = 50 V to V_G = -50 V. When nitrogen purging is off and oxygen is present around the metasurface, the modulation range decreases rapidly after a few cycles. When nitrogen purging is on, oxygen is removed around the metasurface preventing the graphene to be burnt out, and a modulation range is maintained for many modulation cycles.

11.2 Dirac voltage stabilization during the nitrogen purging

When the nitrogen purging is started, the Dirac voltage of the graphene marginally shifts during the first few gate bias cycles. This is presumed to be due to the adsorbates, which are adsorbed on the graphene when it is exposed to the air, detaching during the nitrogen purging and gate bias cycles[19]. Figure S17b shows the overall shift in the sheet resistance-gate bias curve from the start of nitrogen purging until the Dirac voltage stabilizes. At both end gate biases of V_G = -80 V and V_G = 70 V (Fig. S17c), graphene sheet resistance converges to a certain value and stabilizes over the cycle. All measurements in this experiment are conducted after confirming this stabilization.

Supplementary Note 12. Material refractive index fitting

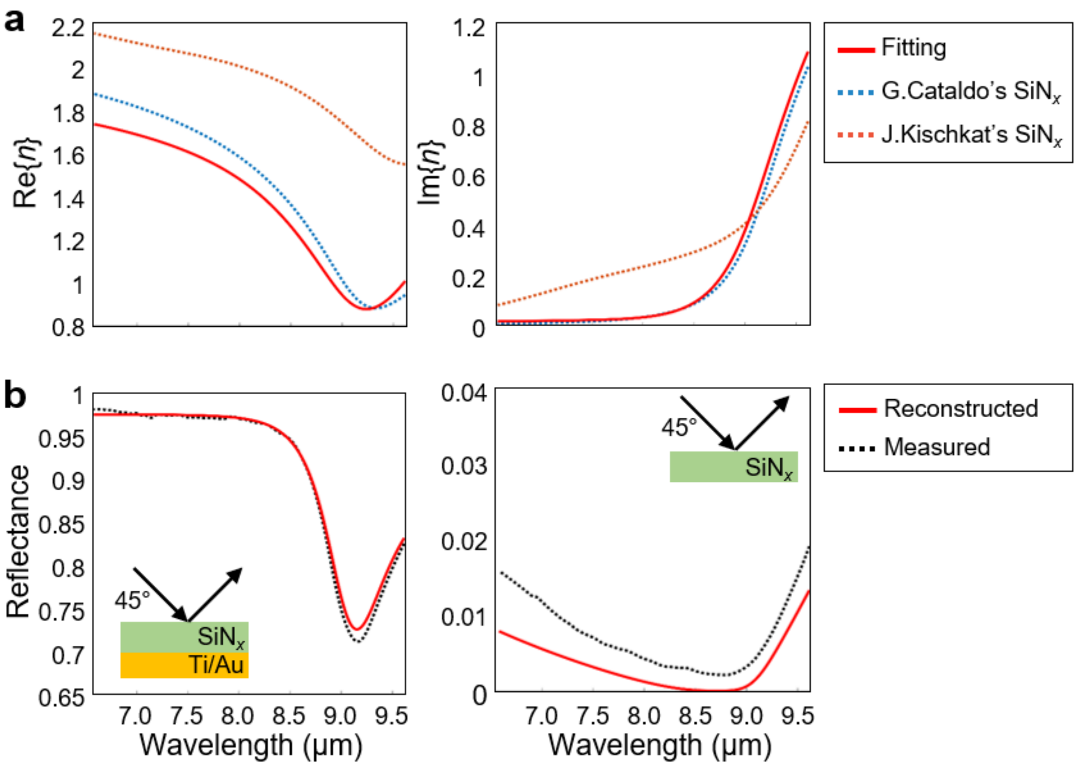

Fig. S18. Results of fitting the refractive index of low-stress silicon nitride. a Real part (left panel) and imaginary part (right panel) of the fitted refractive index of low-stress silicon nitride (red solid line) as a function of wavelength. For comparison, the refractive index data of silicon nitride from G. Cataldo[22] (blue dashed line) and J. Kischkat[23] (red dashed line) are also plotted. **b** Reconstructed (red solid line) and measured (black dashed line) reflection spectra of the silicon nitride membrane with (left panel) and without (right panel) the deposition of a back reflector.

The refractive index of the low-stress silicon nitride used in the device is determined through reflectance measurements, utilizing the same optical setup illustrated in Fig. 2a. A 200 nm-thick silicon nitride membrane is measured both with and without the deposition of a 3 nm Ti adhesion layer and a 70 nm gold back reflector. TM-polarized light at an incidence angle of 45° is used to measure reflectance over the wavelength range of 6.589 µm to 9.615 µm. The dispersions of the refractive index (n) and relative permittivity (ε) are fitted to the two measured reflectance spectra using the Brendel-Bormann model, as shown in Eq. S16-S19[20]:

$$\varepsilon(\nu) = \varepsilon_\infty + \sum_{k=1}^{m} \chi_k(\nu), \tag{S16}$$

$$\chi_k(\nu) = \frac{i\sqrt{\pi}\,\nu_{pk}^2}{2\sqrt{2}a_k(\nu)\sigma_k}\left[W\left(\frac{a_k(\nu)-\nu_{0k}}{\sqrt{2}\sigma_k}\right)+W\left(\frac{a_k(\nu)+\nu_{0k}}{\sqrt{2}\sigma_k}\right)\right] \tag{S17}$$

where

$$W(x) = e^{-x^2}\left(1 + \frac{2i}{\sqrt{\pi}}\int_0^x e^{t^2}dt\right), \tag{S18}$$

$$a_k(\nu) = \sqrt{\nu^2 + i\nu_{\tau k}\nu}. \tag{S19}$$

Transfer-matrix method (TMM) is used to obtain theoretical reflectance spectra[21]. The detailed parameter values used for fitting to the Brendel-Bormann model are shown in Table S1.

ε_∞	ν_{p1}	ν_{01}	$\nu_{\tau 1}$	σ_1	ν_{p2}	ν_{02}	$\nu_{\tau 2}$	σ_2
3.753	1056	880.0	5.141	108.8	779.2	778.5	6715	1330

Table S1. Values of the Brendel-Bormann model parameters. All parameters except for ε_∞ are in cm^{-1}.

The fitted dispersion of the refractive index of low-stress silicon nitride is shown in Fig. S18a, compared with the results obtained by G. Cataldo[22] and J. Kischkat[23]. The reconstructed reflectance spectra using the fitted refractive index of low-stress silicon nitride show good agreement with the measured spectra in both cases, as shown in Fig. S18b. For the purpose of complex frequency analysis, the complex frequency is substituted into the frequency term in Eq. S16-S19.

Supplementary Note 13. Angular divergence of the reflected beam

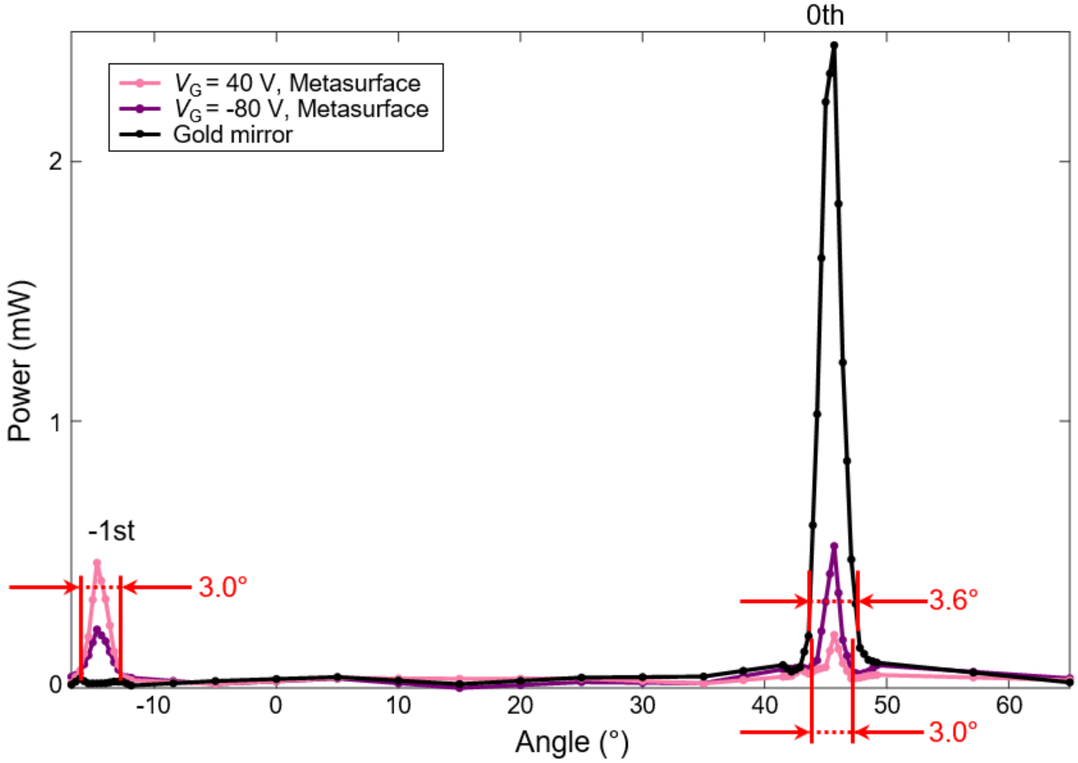

Fig. S19. Angular radiation pattern of the reflected beam from the gold mirror (black line) and the metasurfaces at V_G = 40 V (pink line) and V_G = -80 V (purple line). The angular divergence of main lobes of each angular radiation pattern is denoted.

To measure the angular divergence of the reflected beam of the 0th and the -1st order diffraction, the powermeter doesn't have sufficient angular resolution because it collected data in the angular range of $\Delta\theta = 7.125°$ at once. By placing a narrow slit in front of the powermeter, the angular resolution is increased to $\Delta\theta = 0.36°$. The angular divergence is measured at a frequency f_0 = 37.05 THz (λ_0 = 8.092 μm) with a metasurface different from the one shown in the main paper due to their different structure parameters. The size of the entire metasurface is the same as the metasurface in the main paper, and the beam spot diameter is $2w_0$ = 199 μm (w_0 is the beam waist.), which is smaller than the size of the entire metasurface. The angular divergence of a peak Θ is defined as the full angular width at the point where the reflected power is $1/e^2$ of the maximum reflected power of that peak, which is expressed as $\Theta = 2\lambda_0/(\pi w_0)$ approximately[24]. In our experimental setup, it is calculated to be 2.97° for an idealized Gaussian beam. Figure S19 presents the measured angular radiated power. For the specularly reflected beam from the gold mirror, the angular divergence is measured to be 3.6°. For the beam reflected from the metasurface, the main lobe presents an angular divergence of 3.0° when the gate bias is V_G = -80 V, and 3.0° when V_G = 40 V. The measured beams show marginal deviations from the ideally calculated values. This discrepancy can be attributed

to spatial inhomogeneity of structure, imperfect alignment of the optical setup, and imperfect incident laser beam quality, which can slightly distort the wavefront of the reflected beam to be deviated from the ideal Gaussian beam.

Supplementary Note 14. Simulated analysis on the operating bandwidth

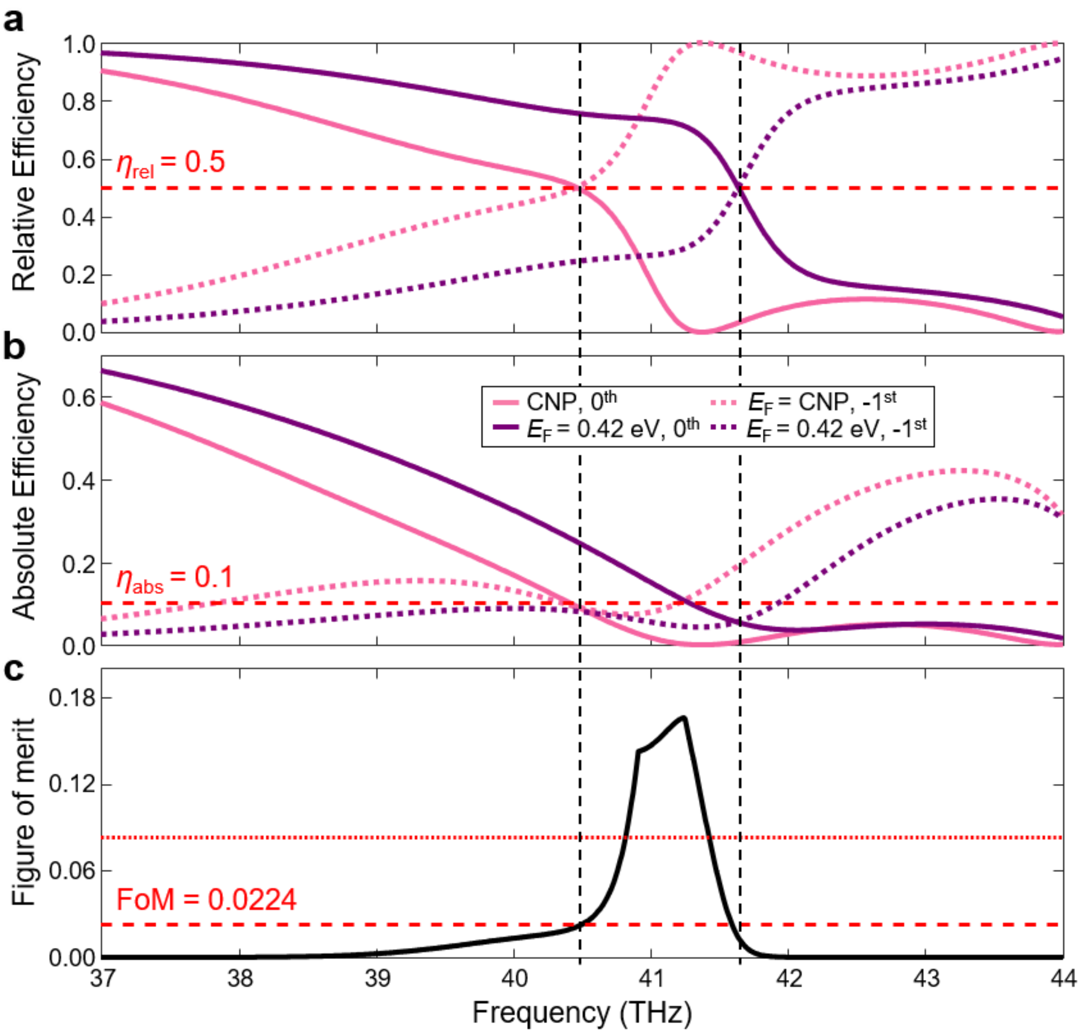

Fig. S20. Calculated **a** relative efficiency, **b** absolute efficiency, and **c** figure of merit spectra of the fabricated device. In between two vertical dashed lines, active beam switching behavior appears. The threshold line of each spectrum is drawn as a red dashed line.

At a graphene carrier mobility of 200 cm^{2}V^{-1}s^{-1}, the absolute efficiency, relative efficiency, and figure of merit (FoM) spectra of our fabricated metasurfaces are shown in Fig. S20. To determine the frequency bandwidth over which beam switching occurs, the relative efficiency of each target diffraction order at its corresponding Fermi level must be $\eta_{rel} \geq 0.5$. In Fig. S20a, the relative efficiency spectra for each Fermi level and diffraction order are plotted, with the threshold line $\eta_{rel} = 0.5$ shown as a red dashed horizontal line. For our fabricated device, the 0th order diffraction is maximized at the graphene charge neutrality point (CNP), while the -1st order is maximized at $E_F = 0.42$ eV. (1) The corresponding spectra, shown as purple solid and pink dashed lines, should be above the threshold line. (2) Simultaneously, the purple dashed and pink solid lines represent the off-target

diffraction orders, which should remain below this threshold. The beam-switching bandwidth that satisfies both (1) and (2) is demarcated by the vertical black dashed lines and spans 1.14 THz.

Compared to conventional electro-optic beam switching devices, the absolute efficiency above 0.1 (10%) marks a notable improvement over previous studies[25-29]. In Fig. S20b, within the beam switching bandwidth, the absolute efficiencies of both the purple solid line and the pink dashed line have values near this $\eta_{abs} = 0.1$ threshold. To consider both absolute and relative efficiencies, we set the figure of merit threshold at FoM = 0.0224 drawn as a red dashed line in Fig. S20c. This threshold value is derived from the absolute ($\eta_{abs} = 0.1$) and the relative ($\eta_{rel} = 0.5$) efficiency thresholds. The frequency bandwidth over which the FoM is above the threshold is 1.11 THz. On the other hand, the simple full width at half maximum (FWHM) calculation of the FoM yields 0.61 THz (red dotted line in Fig. S20c).

Supplementary Note 15. Three-level beam switching metasurface with ionic gating

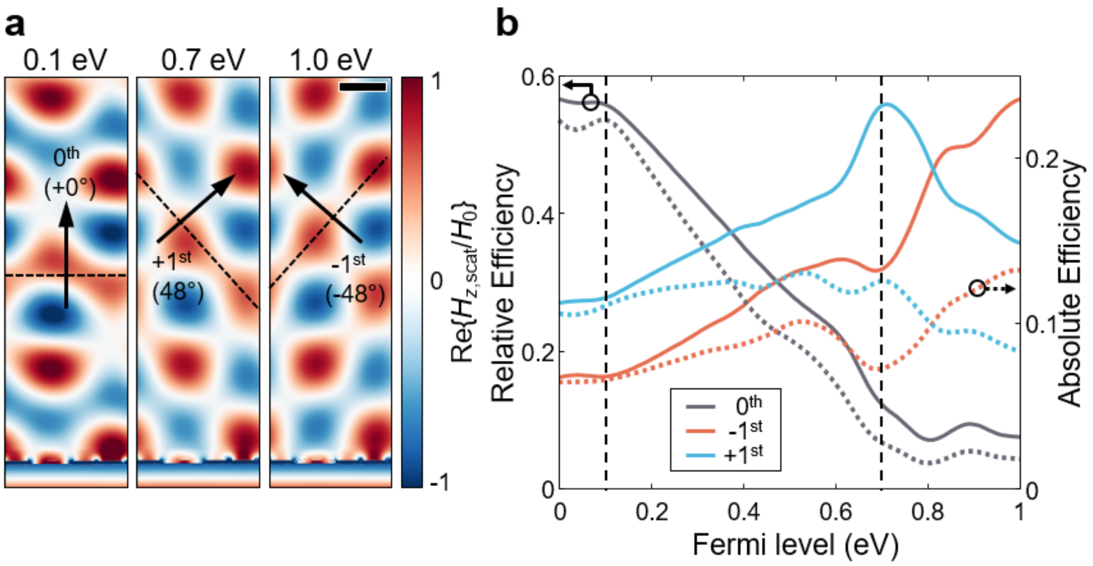

Fig. S21. a Simulated scattered magnetic field profiles of ionic gel type three-level beam switching device. **b** Its beam switching performance as a function of Fermi level. Solid lines represent the relative efficiency (left axis) and dotted lines represent the absolute efficiency (right axis). The scale bars are 4 μm.

Because adding the ionic gel layer can affect the beam switching performance, we investigated whether beam switching remains appreciable under the ionic gating scheme. Figure S21 indicates that three-level beam switching is theoretically achievable even when a 1 μm ionic gel layer is introduced on top of the metasurface. If the graphene Fermi level is raised to 0.1 eV, 0.7 eV, and 1.0 eV via ionic gating, the scattered magnetic field is directed to the 0th, +1st, and -1st order, respectively, showing that three-level switching is still possible. In this simulation, the refractive index of the ionic gel was measured using a mid-infrared spectroscopic ellipsometry.

Supplementary Note 16. Possible applications of the platform

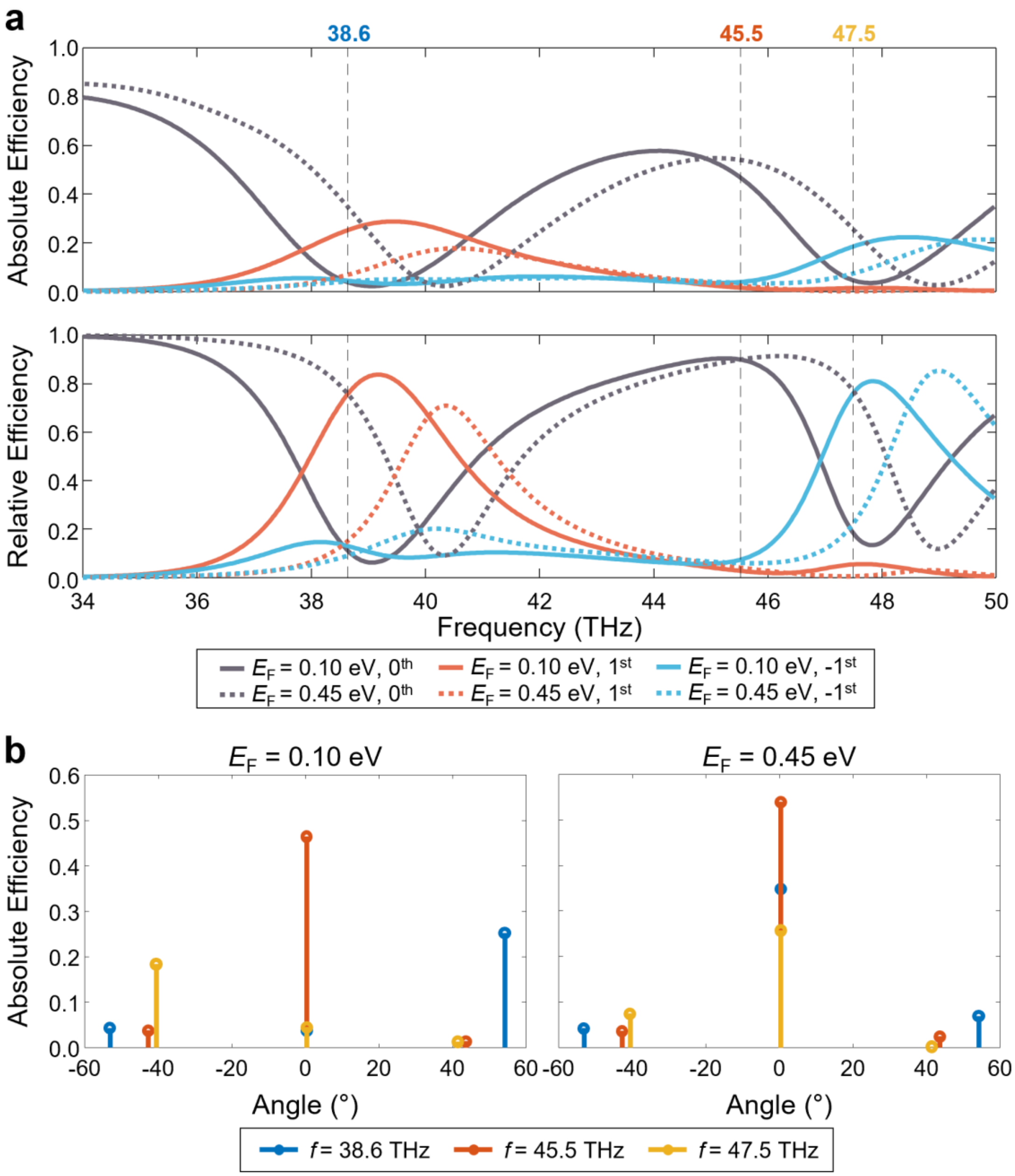

Fig. S22. a Simulated absolute (top) and relative (bottom) efficiencies of the 0th, +1st, and **-**1st reflection orders as a function of frequency under two graphene Fermi levels: E_F = 0.10 eV (solid lines) and E_F = 0.45 eV (dotted lines). Three representative target frequencies (38.6, 45.5, and 47.5 THz) are indicated by vertical dashed vertical lines. **b** Absolute efficiency versus diffraction angle for the three target frequencies, shown for both Fermi levels. At E_F = 0.10 eV, different frequencies are diffracted into distinct angles (demultiplexed), whereas at E_F = 0.45 eV, all frequencies are predominantly reflected into the 0th order (recombined).

16.1 Freespace tunable beam splitter

Tunable beam splitters are essential components in modern quantum optics, particularly in quantum computation[30], quantum metrology[31], and quantum simulation[32]. In many of these systems, the beam splitting ratio must be dynamically controlled to manipulate quantum states or to implement reconfigurable quantum gates. Conventionally, this has been realized in integrated photonic circuits using Mach-Zehnder interferometers with electro-optic phase shifters[33]. While these platforms offer high stability and scalability in chip-scale environments, their integration often limits the interaction to waveguide-bound geometries, thereby constraining their flexibility in broader optical architectures, especially in freespace settings.

In contrast, our proposed metasurface provides a compelling route toward freespace tunable beam splitter which can continuously adjust splitting ratio (relative efficiency) with applied single-gate bias. Comparing the traditional types, it offers ultra-thin form factor, low power consumption, and potential for high-speed modulation. The demonstrated beam switching between the 0th and -1st diffraction orders (Fig. 2b-d in the manuscript), with nearly symmetric absolute efficiencies and a wide angular separation (57°), is directly analogous to a 1×2 beam splitter with a dynamically tunable splitting ratio. Such functionality is particularly attractive for quantum photonic experiments that require rapid, non-mechanical control of spatial optical paths. This makes it well-suited for future integration with cryogenic setups or compact freespace quantum modules.

16.2 Freespace tunable beam demultiplexer

This device platform can also operate as a freespace tunable beam demultiplexer, where multiple frequency components are spatially separated or recombined in response to a gate bias. This type of functionality is particularly relevant for spectrally multiplexed communication systems, adaptive spectroscopy, or optical routing, where dynamic control over frequency-to-angle mapping is needed[34].

In Fig. S22, we show the simulated optical efficiencies spectra of the 0th, +1st, and -1st diffraction orders under two representative graphene Fermi levels: 0.10 eV (solid lines) and 0.45 eV (dotted lines), with normal incidence. The optimized structure has gold strip width w_i = (1294, 1861, 1350, 1915, 196) nm, gap g_i = (2245, 275, 98, 46, 348) nm, gold strip height h = 33.5 nm, and a graphene carrier mobility of 1,000 $cm^2V^{-1}s^{-1}$. Target frequencies 38.6 THz, 45.5 THz and, 47.5 THz are chosen to show freespace tunable beam demultiplexing behavior. Figure S22b depicts absolute efficiency versus diffraction angle for the three target frequencies at both Fermi levels. At E_F = 0.10 eV of graphene Fermi level, distinct frequency components are each scattered into separate diffraction orders and angles, effectively splitting an incident multi-frequency beam into spatially resolved output channels. This corresponds to a demultiplexing mode, where input frequencies are directed to different angular positions. In contrast, when the Fermi level is increased to E_F = 0.45 eV, the device response is dominated by the 0th order diffraction for all three frequencies, with minimal angular dispersion. This state corresponds to a recombined or pass-through mode, where the incident light is redirected nearly entirely into the

specular direction. This electro-optic switching between demultiplexing and recombining behaviors, achieved via a single gate bias, demonstrates the potential of our device to operate as a tunable frequency-to-angle mapping platform in freespace. The ability to perform this control without mechanical components or multi-electrode addressing represents a significant step toward compact, scalable optical signal processors in mid-infrared applications.

References

1. Siegel, J., et al. Electrostatic steering of thermal emission with active metasurface control of delocalized modes. *Nat. Commun.* **15**, 3376 (2024).

2. Wang, H., et al. Normally-off fully recess-gated GaN metal–insulator–semiconductor field-effect transistor using Al2O3/Si3N4 bilayer as gate dielectrics. *Appl. Phys. Express* **10**, 106502 (2017).

3. Kim, S., et al. Realization of a high mobility dual-gated graphene field-effect transistor with Al2O3 dielectric. *Appl. Phys. Lett.* **94**, 6 (2009).

4. Hugonin, J. P. & Lalanne, P. Reticolo software for grating analysis. Preprint at https://arxiv.org/abs/2101.00901 (2021).

5. Lalanne, P. & Morris, G. M. Highly improved convergence of the coupled-wave method for TM polarization. *J. Opt. Soc. Am. A* **13**, 779-784 (1996).

6. Mitchell, M. *An Introduction to Genetic Algorithms* (MIT Press, 1998).

7. Holland, J. H. Genetic algorithms. *Sci. Am.* **267**, 66-73 (1992).

8. Mooney, C. Z. *Monte Carlo Simulation* (Sage, 1997).

9. Chang, T. H. P. Proximity effect in electron-beam lithography. *J. Vac. Sci. Technol.* **12**, 1271-1275 (1975).

10. Chung, H. & Miller, O. D. High-NA achromatic metalenses by inverse design. *Opt. Express* **28**, 6945-6965 (2020).

11. Yu, N., et al. Light propagation with phase discontinuities: Generalized laws of reflection and refraction. *Science* **334**, 333-337 (2011).

12. Meade, R. D., Johnson, S. G. & Winn, J. N. *Photonic Crystals: Molding the Flow of Light* (Princeton Univ. Press, 2008).

13. Kim, J. Y., et al. Full 2π tunable phase modulation using avoided crossing of resonances. *Nat. Commun.* **13**, 2103 (2022).

14. Kim, S., et al. Electronically tunable perfect absorption in graphene. *Nano Lett.* **18**, 971-979 (2018).

15. Kim, S., et al. Electronically tunable extraordinary optical transmission in graphene plasmonic ribbons coupled to subwavelength metallic slit arrays. *Nat. Commun.* **7**, 12323 (2016).

16. Han, S., et al. Complete complex amplitude modulation with electronically tunable graphene plasmonic metamolecules. *ACS Nano* **14**, 1166-1175 (2020).

17. Betz, F., Binkowski, F. & Burger, S. RPExpand: Software for Riesz projection expansion of resonance phenomena. *SoftwareX* **15**, 100763 (2021).

18. Binkowski, F., Zschiedrich, L. & Burger, S. A Riesz-projection-based method for nonlinear eigenvalue problems. *J. Comput. Phys.* **419**, 109678 (2020).

19. Kong, L., et al. Molecular adsorption on graphene. *J. Phys. Condens. Matter* **26**, 443001 (2014).

20. Brendel, R. & Bormann, D. An infrared dielectric function model for amorphous solids. *J. Appl. Phys.* **71**, 1-6 (1992).

21. Katsidis, C. C. & Siapkas, D. I. General transfer-matrix method for optical multilayer systems with coherent, partially coherent, and incoherent interference. *Appl. Opt.* **41**, 3978-3987 (2002).

22. Cataldo, G., et al. Infrared dielectric properties of low-stress silicon nitride. *Opt. Lett.* **37**, 4200-4202 (2012).

23. Kischkat, J., et al. Mid-infrared optical properties of thin films of aluminum oxide, titanium dioxide, silicon dioxide, aluminum nitride, and silicon nitride. *Appl. Opt.* **51**, 6789-6798 (2012).

24. Saleh, B. E. A. & Teich, M. C. *Fundamentals of Photonics* (John Wiley & Sons, 2019).

25. Li, M., et al. Excitonic beam steering in an active van der Waals metasurface. *Nano Lett.* **23**, 2771-2777 (2023).

26. Park, J., et al. All-solid-state spatial light modulator with independent phase and amplitude control for three-dimensional LiDAR applications. *Nat. Nanotechnol.* **16**, 69-76 (2021).

27. Karst, J., et al. Electrically switchable metallic polymer nanoantennas. *Science* **374**, 612-616 (2021).

28. Shirmanesh, G. K., et al. Electro-optically tunable multifunctional metasurfaces. *ACS Nano* **14**, 6912-6920 (2020).

29. Thureja, P., et al. Array-level inverse design of beam steering active metasurfaces. *ACS Nano* **14**, 15042-15055 (2020).

30. Knill, E., et al. A scheme for efficient quantum computation with linear optics. *Nature* **409**, 46–52 (2001).

31. Higgins, B. L., et al. Entanglement-free Heisenberg-limited phase estimation. *Nature* **450**, 393–396 (2007).

32. Lanyon, B. P., et al. Towards quantum chemistry on a quantum computer. *Nat. Chem.* **2**, 106–111 (2010).

33. Ma, X. S., et al. A high-speed tunable beam splitter for feed-forward photonic quantum information processing. *Opt. Express* **19**, 22723–22730 (2011).

34. Ma, J., et al. Frequency-division multiplexer and demultiplexer for terahertz wireless links. *Nat. Commun.* **8**, 729 (2017).
